# Personalization of Logical Models With Multi-Omics Data Allows Clinical Stratification of Patients

**DOI:** 10.3389/fphys.2018.01965

**Published:** 2019-01-24

**Authors:** Jonas Béal, Arnau Montagud, Pauline Traynard, Emmanuel Barillot, Laurence Calzone

**Affiliations:** Institut Curie, PSL Research University, Mines Paris Tech, Inserm, U900, Paris, France

**Keywords:** logical models, personalized mechanistic models, personalized medicine, breast cancer, data discretization, stochastic simulations

## Abstract

Logical models of cancer pathways are typically built by mining the literature for relevant experimental observations. They are usually generic as they apply for large cohorts of individuals. As a consequence, they generally do not capture the heterogeneity of patient tumors and their therapeutic responses. We present here a novel framework, referred to as PROFILE, to tailor logical models to a particular biological sample such as a patient tumor. This methodology permits to compare the model simulations to individual clinical data, i.e., survival time. Our approach focuses on integrating mutation data, copy number alterations (CNA), and expression data (transcriptomics or proteomics) to logical models. These data need first to be either binarized or set between 0 and 1, and can then be incorporated in the logical model by modifying the activity of the node, the initial conditions or the state transition rates. The use of MaBoSS, a tool based on Monte-Carlo kinetic algorithm to perform stochastic simulations on logical models results in model state probabilities, and allows for a semi-quantitative study of the model phenotypes and perturbations. As a proof of concept, we use a published generic model of cancer signaling pathways and molecular data from METABRIC breast cancer patients. For this example, we test several combinations of data incorporation and discuss that, with these data, the most comprehensive patient-specific cancer models are obtained by modifying the nodes' activity of the model with mutations, in combination or not with CNA data, and altering the transition rates with RNA expression. We conclude that these model simulations show good correlation with clinical data such as patients' Nottingham prognostic index (NPI) subgrouping and survival time. We observe that two highly relevant cancer phenotypes derived from personalized models, *Proliferation* and *Apoptosis*, are biologically consistent prognostic factors: patients with both high proliferation and low apoptosis have the worst survival rate, and conversely. Our approach aims to combine the mechanistic insights of logical modeling with multi-omics data integration to provide patient-relevant models. This work leads to the use of logical modeling for precision medicine and will eventually facilitate the choice of patient-specific drug treatments by physicians.

## 1. Introduction

Molecular profiling of patient samples is now becoming clinical routine in diseases like cancer, where it has shown therapeutic utility. Typically, tumor DNA or RNA are sequenced, and if an oncogene mutation is found, then it opens the opportunity to treat the patient with a targeted inhibitory drug which counteracts the mutated oncoprotein effect. Nevertheless, this strategy has often limited impact, because the tumor will eventually activate compensatory pathways or acquire novel mutations and escape the treatment. To anticipate drug resistance and optimize treatments, a better understanding of the regulatory network dynamics is needed. As a consequence, mathematical modeling has been increasingly used to formally describe the dynamics of regulatory networks representing the signaling pathways that are frequently altered in cancers. Many of these signaling pathways, e.g., apoptosis, mTOR pathway, RTK signaling, or DNA repair pathways, are shared among diverse cancers and contain common mutations or gene alterations. The translation of the networks recapitulating these pathways into mathematical models can be done using different formalisms. Over the past decades, numerous uses of logical modeling have shown that this framework is able to characterize the main dynamical properties of complex biological regulatory networks (Faure et al., 2006; Abou-Jaoudé et al., 2011; Grieco et al., 2013), as well as to predict the behavior of molecular networks affected in human diseases (Fumiã and Martins, 2013; Arshad and Datta, 2017).

However, these models usually describe general processes and tend to be generic, missing patients' specificities and possible patient-tailored interventions. To avoid the relapse that follows many treatments, these models need to be adjusted to each individual patient, capitalizing on omics profile of the patient tumor. Some work has been done on trying to contextualize these models to perturbation data (often (phospho-)proteomics data) (Saez-Rodriguez et al., 2009; Rodriguez et al., 2015; Dorier et al., 2016) but it remains difficult to apply these methods to patient data (typically genome and transcriptome) and get clinical insight. Additionally, some network-based methods have been investigated for patient stratification, using network propagation with somatic mutations (Hofree et al., 2013) or applying propagation of gene expression data on KEGG pathways coupled with mutation information (Hidalgo et al., 2017).

**Figure 1:**
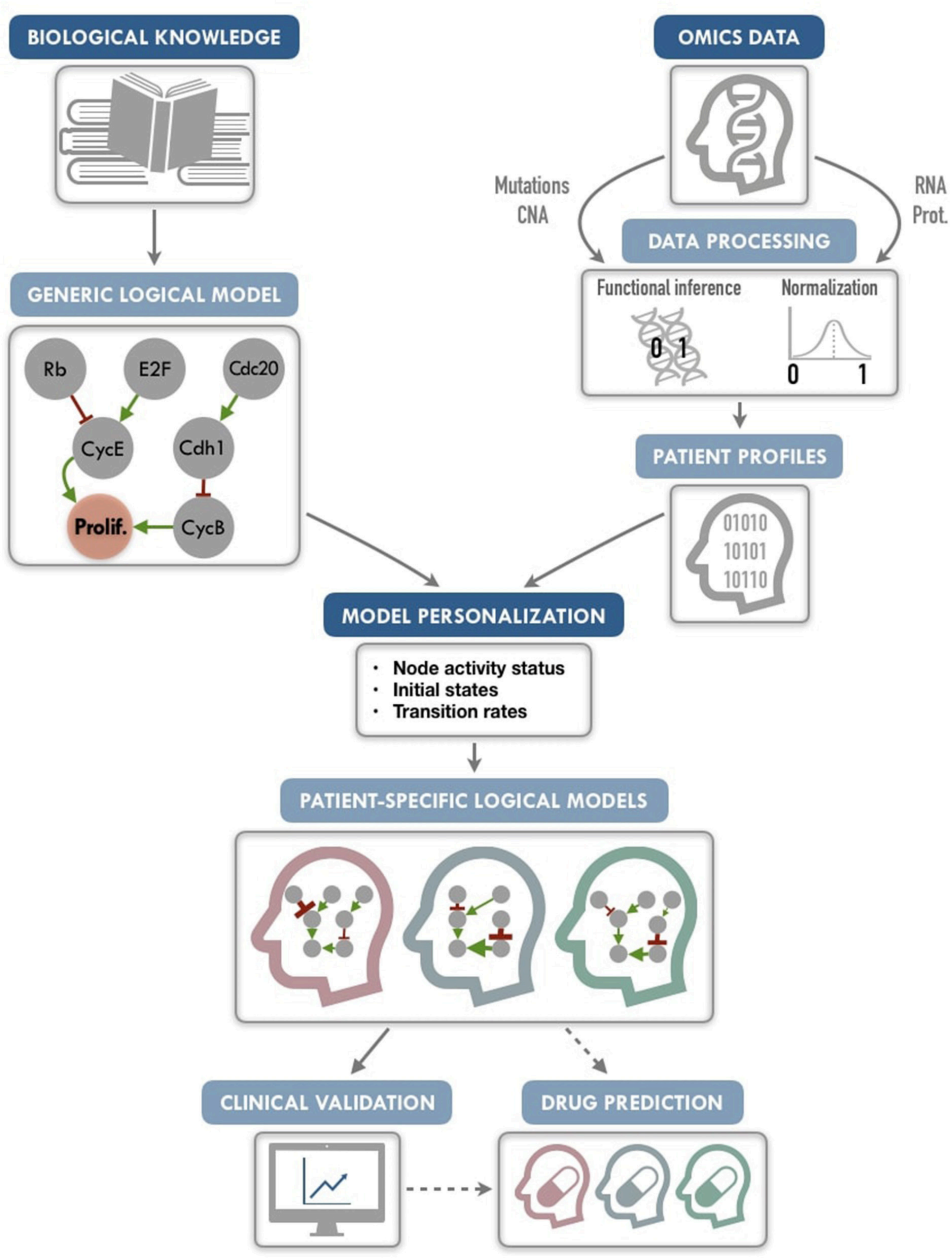
PROFILE methodology for personalization of logical models. On the one hand (upper left), a generic logical model, in a MaBoSS format (a BND file for model description with logical rules and a CFG file for definition of the simulation parameters), is selected to serve as the starting-point. Note that any SBML qual model can be easily translated into a MaBoSS format. The parameters related to the nodes (initial states and transition rates) are chosen to be generic in the initial CFG file. On the other hand (upper right), omics data are gathered (e.g., genome and transcriptome) as data frames, and processed through functional inference methods (for already discrete genome data) or binarization/normalization (for continuous expression data). The resulting patient profiles are used to perform model personalization, i.e., adapt the generic model with patient data. The merging of the generic model with the patient profiles creates a personalized MaBoSS model with an unchanged BND file and a CFG file per patient. Then, clinical relevance of these patient-specific models can be assessed before providing original and personalized therapeutic strategies and drug predictions.

Our PROFILE (**P**e**R**sonalization **OF** log**I**ca**L** Mod**E**ls) approach aims to combine the mechanistic insights of logical modeling with multi-omics data integration to provide patient-relevant models (Figure 1). The generic logical model can be any model in standard format, automatically translated into a format specific to MaBoSS (**Ma**rkovian **Bo**olean **S**tochastic **S**imulator), a tool that simulates continuous time Markov processes on Boolean networks (Stoll et al., 2012, 2017). The biological data are extracted from existing repositories or from private sources into a data frame per data type. The merging of these two inputs provides a personalized logical model per patient. Therefore, we define a personalization of a logical model as a specification of a generic logical model using available patient data. We present here a framework to tailor a logical model to patient-specific multi-omics data, thereby personalizing these generic models to particular patients or sets of patients with the goal to treat these patients in a personalized manner. We also show how to best use mutation, copy number and transcriptome patient data for model personalization. To illustrate the method, we gathered 2,509 breast cancer data genomic profiles from METABRIC project, including somatic mutations, copy number alterations, and gene expression (Curtis et al., 2012; Pereira et al., 2016), and integrated the data on a published logical model of generic cancer pathways (Fumiã and Martins, 2013) using MaBoSS. Lastly, we show evidence that our patient-specific models can be used to stratify patients by groups and by survival data.

We conclude that this framework allows us to provide models that can capture detailed descriptions of patient data, paving the way to modeling patient response to many potential targeted treatments or combination of treatments, and helping the clinical oncologists to choose the best option for personalized treatment (Figure 1). The framework can be used on any logical model, available in databases such as Cell Collective (https://cellcollective.org), and with any set of patient data, and thus used by non-experts in modeling.

It is freely available on GitHub (https://github.com/sysbio-curie/PROFILE) and is distributed open source under the BSD 3-clause license.

## 2. Materials and Methods

### 2.1. Logical Modeling

#### 2.1.1. Principles

Although continuous mathematical modeling based on chemical kinetics has been widely used to study cellular biochemistry dynamics (e.g., ordinary differential equations) (Novák and Tyson, 2004; Fey et al., 2015), this formalism faces limits for modeling large-scale signaling networks, due to the difficulty of estimating kinetic parameter values. In contrast, the logical modeling formalism represents a convenient mean of abstraction, where the causal relationships between proteins (or genes) are encoded with logical statements and dynamical behaviors are represented by transitions between discrete states of the system. The logical formalism is flexible, requires in principle no quantitative information, and, hence, can be applied to large networks combining multiple pathways. It can also provide a qualitative understanding of molecular systems lacking mechanistic detailed information. A brief summary of the main features of logical modeling is provided hereunder and a more detailed primer can be found in Supplementary Material. For more in-depth reviews on logical models, their construction and analyses, we refer the reader to several sources (Saadatpour and Albert, 2013; Le Novère, 2015; Abou-Jaoudé et al., 2016).

**Figure 2:**
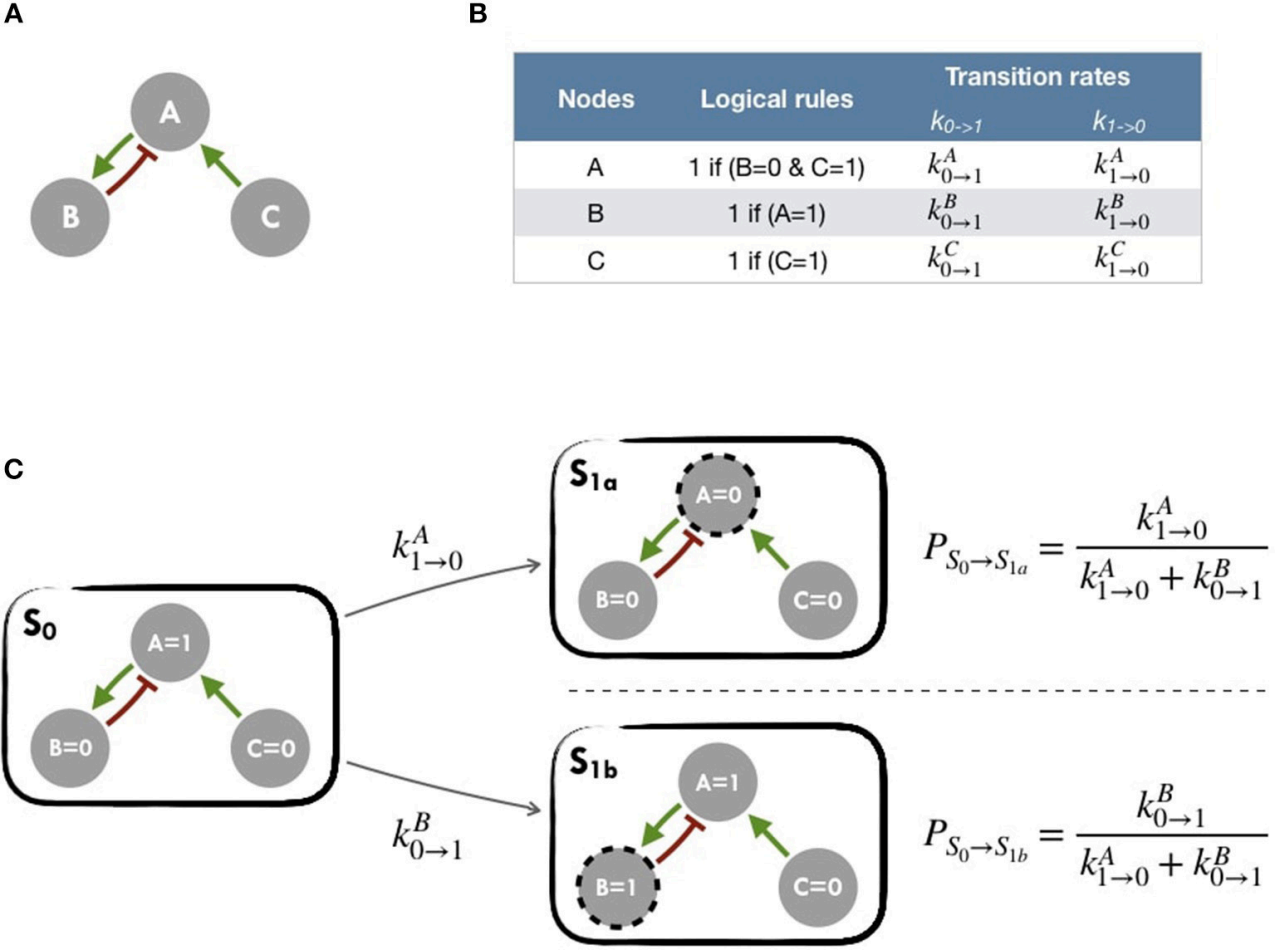
Main principles of MaBoSS simulation framework and Gillespie algorithm **(A)** Toy model with B and C regulated by A. **(B)** In the first column, logical rules of the logical model: as an input A remains in its initial state; presence of A triggers B activation and C inhibition. In the second column, MaBoSS activation and inactivation transition rates are defined for each possible transition **(C)** In an asynchronous update scheme, starting from *S*_0_ state, there are two possible following states *S*_1*a*_ and *S*_1*b*_ with their corresponding probabilities.

A logical model is based on a regulatory graph, where each node represents a component (e.g., a protein, gene, complex, process, etc.), and is associated with discrete levels of activity (0, 1, or more when justified) as represented in Figure 2A. Each edge corresponds to a regulatory interaction between the source and target nodes, and is represented by a positive or negative influence, depending on the type of regulation. Logical rules (or functions) are assigned to each node of the network. These rules connect input nodes with logical operators AND (&), OR (|) and NOT (!), or a combination of these operators (Figure 2B). An example of a toy model can be found in Figure 2A and Figure S1.

The resulting dynamics can be represented in terms of a second type of graphs, the state transition graph (STG), where the nodes account for the states of the system, referred to as the model states (Figure 2C). The model states correspond to vectors of the nodes' activity, and the edges to the possible state transitions from one model state to another. When concurrent variable changes are enabled at a given state, the resulting state transition depends on the chosen updating assumption. Numerous studies use the simple fully synchronous strategy where all variables are updated through a unique transition (Weinstein et al., 2017). This assumption leads to relatively simple STG and deterministic dynamics (Helikar et al., 2008; Fumiã and Martins, 2013; Cho et al., 2016). However, the synchronous updating assumption may lead to spurious cyclic attractors. The asynchronous updating strategy considers separately all possible transitions and therefore provides alternative dynamics in the absence of kinetic data. The resulting dynamics have a branching structure that complicates its evaluation. An example of such graphs can be found in Figure 2C or Figure S2 for an asynchronous graph and Figure S3 for a synchronous graph.

In this work, asynchronous dynamics with stochastic simulations have been considered.

More details of logical models and their uses can be found in other works such as Abou-Jaoudé et al. (2016) and Chaouiya et al. (2012).

#### 2.1.2. Simulations With MaBoSS

MaBoSS software is applied to obtain probabilities for each of the model states of the system using continuous time Markov chain simulations on the Boolean network (Stoll et al., 2012, 2017). Its principles are summarized in Figure 2 and in Figure S5 for a more comprehensive version. MaBoSS uses a specific language for associating transition rates, *k*_0→1_ (or *k*_*up*_) and *k*_1→0_ (or *k*_*down*_), to each node (Figure 2B), enabling to account for different time scales of the processes described by the model. Given some initial conditions (i.e., either 0 or 1 state for each node), MaBoSS applies Monte-Carlo kinetic algorithm (or Gillespie algorithm) to the network.

This algorithm provides a stochastic way to choose a specific transition among several possible ones (Figure 2C), to perform asynchronous updates and finally to infer a corresponding time for this transition (Figure S5D). Thus, by concatenating stochastic updates, MaBoSS computation results in one stochastic trajectory as a function of time. The transition rates can be understood as probabilities in order to determine the actual transition. For our simulations, unless otherwise specified, all transition states were initially assigned to 1. Since MaBoSS computes stochastic trajectories, it is relevant to generate a population of stochastic trajectories to gain insight into the average behavior over the asynchronous STG.

The aggregation of stochastic trajectories can also be interpreted as a description of an heterogeneous population. Since several trajectories are simulated, initial values of each node can be defined with a continuous value between 0 and 1 representing the probability for the node to be defined to 1 for each new trajectory. For instance, a node with a 0.6 initial condition will be set to 1 in 60% of simulated trajectories and to 0 in 40% of them.

Two files are needed to run MaBoSS: a model file (BND) where the nodes of the model and their logical rules are listed and a configuration file (CFG) where initial states, transition rates and other parameters of the simulation are specified.

In the present work, all simulations were performed with MaBoSS and the focus has been set on the probabilities of nodes and phenotypes at the asymptotic state. Indeed, asymptotic states are more closely related to logical model attractors than transient dynamics. They are therefore less dependent on updating stochasticity and are more meaningful biologically (Huang et al., 2009).

Only 1,000 stochastic trajectories were computed in all simulations since it appeared as a sufficient number to obtain a median standard deviation below 0.01 (see Figure S9). For any study using MaBoSS, to insure that the state space is well explored, it is advised to start with a higher number of trajectories at first and reduce it when the median deviation is below a reasonable threshold.

Examples of MaBoSS applied to biological questions can be found in Calzone et al. (2010); Cohen et al. (2015); Remy et al. (2015); or Montagud et al. (2017). Any logical model in SBML qual format (Chaouiya et al., 2013) can be exported from GINsim (Chaouiya et al., 2012) into MaBoSS format, allowing the use of any logical model from databases for the PROFILE framework.

#### 2.1.3. Generic Logical Model of Cancer Pathways

A published Boolean network model was used to illustrate our PROFILE methodology (Fumiã and Martins, 2013). It is based on a regulatory network summarizing several key players and pathways involved in cancer mechanisms: RTKs, PI3K/AKT, WNT/β-catenin, TGF-β/Smads, Rb, HIF-1, p53 and ATM/ATR. An input node *Acidosis* and an output node *Proliferation* used as a read-out were added to ease the analysis. Based on the model's logical rules from Fumiã and Martins (2013), *Proliferation* node is activated by any of the cyclins (*CyclinA, CyclinB, CyclinD*, and *CyclinE*) and is, thus, an indicator of cyclin activity as an abstraction of the cell cycle behavior. This is a simplification of cell cycle, and if readers would like to go beyond this abstraction, a detailed study on the dynamics of a mammalian cell cycle that takes into account cyclins and cyclin-dependent kinases can be found in Gérard and Goldbeter (2016). The generic model of Fumiã and Martins (2013) contains 98 nodes and 254 edges, and can be visually inspected in Figure S6. It is available in MaBoSS format in our GitHub repository: (https://github.com/sysbio-curie/PROFILE/tree/master/Models/Fumia2013).

### 2.2. Generation of Patient Profiles From Multi-Omics Datasets

#### 2.2.1. TCGA and METABRIC Data

Patient data from METABRIC (Curtis et al., 2012; Pereira et al., 2016) with RNA expression data (*n* = 1,904), mutation profiles (*n* = 2,509), CNA (*n* = 2,173) and clinical data (*n* = 1,980) were gathered. Missing values were considered on an personalization-specific basis: if the personalization method used mutation profiles and RNA data, only the patients with data of these types were considered. More details on the abundance of data types' samples can be found in Figure S11A.

Breast cancer patient data from TCGA (Cancer Genome Atlas Network, 2012; Ciriello et al., 2015) with RNA expression data (*n* = 816), mutation profiles (*n* = 817), CNA (*n* = 816) and clinical data (*n* = 817) were also gathered. For TCGA RNA expression data, data from healthy samples are available (112 samples) along with protein data (RPPA) for 673 patients. More details on the abundance of data types' samples can be found in Figure S11B.

Data were downloaded from cBioPortal^1^ (Gao et al., 2013). To explore all possibilities offered by the two datasets, we have used both of them to show different outcomes, METABRIC results are hereby showcased and TCGA results can be found in Supplementary Material.

### 2.3. Adapting Patient Profiles to a Logical Model

For this analysis, we gathered the following types of data: mutations, copy number variations, transcriptomics, proteomics and clinical data. Usually, mutations and copy number variations can be considered as discrete data and gene or protein expression data as continuous data. Two approaches for handling the data can be used in MaBoSS: (1) discrete data can be directly binarized, and (2) continuous data can either be binarized or normalized (expression values are modified so as to fit between 0 and 1). A logical model is personalized differently according to the type of data used. For instance, a deleterious mutation is integrated into the model by setting the corresponding node to 0 and ignoring the logical rule associated to it. For activating mutation, the node is set to 1. Another approach is to modify the transition rates (speed of activation or inactivation of a node, see section 2.1.2 and Figure S5) according to the impact of the mutation or the level of gene or protein expression (further details in section 2.4).

In many mathematical models related to gene networks, some genes are often listed with a generic name and it is not always clear which gene is responsible of the reaction or if it rather refers to a family of genes (e.g., AKT for AKT1, AKT2, AKT3). Thus, before personalizing the models to patient data, a correspondence between model genes and data must be established and choices must be made on which genes to associate to the model's nodes. For our example, the complete table of correspondence of the model is available in our GitHub repository.

#### 2.3.1. Processing of Discrete Data

Discrete data can be integrated in a straightforward manner through functional inference. From METABRIC database, we gathered mutations and copy number alterations.

##### 2.3.1.1. Mutations

Based on the variant classification provided by the data, inactivating mutations (nonsense, frame-shift insertions or deletions and mutation in splice or translation start sites) are assumed to correspond to loss of function mutations and therefore the corresponding nodes of the model are forced to 0. Missense mutations are matched with OncoKB database (Chakravarty et al., 2017). For each mutation present in the database, an effect is assessed (gain or loss of function assigned to 1 and 0, respectively) with a corresponding confidence based on expert and literature knowledge. Mutations targeting oncogenes (resp. tumor-suppressor genes), as defined in the 2020+ driver gene prediction method (Tokheim et al., 2016), are assumed to be gain of function mutations (resp. loss of function) and therefore assigned to 1 (resp. 0). To rule potential passenger mutations out, each assignment requires a label of deleteriousness either from SIFT (Kumar et al., 2009) or from PolyPhen scores (Adzhubei et al., 2010).

##### 2.3.1.2. Copy number alterations

For CNA integration, only amplifications (+2) and homozygous deletions (–2) (based on GISTIC processing Mermel et al., 2011) are considered, but this choice can be adapted to the focus of the study. Nodes corresponding to amplified genes are set to 1 and those associated with homozygous deletions are set to 0 in patient profiles. In our approach, we chose to discard CNA GISTIC variations with values –1 and +1 due to their low-confidence significance.

#### 2.3.2. Processing of Expression Data

**Figure 3:**
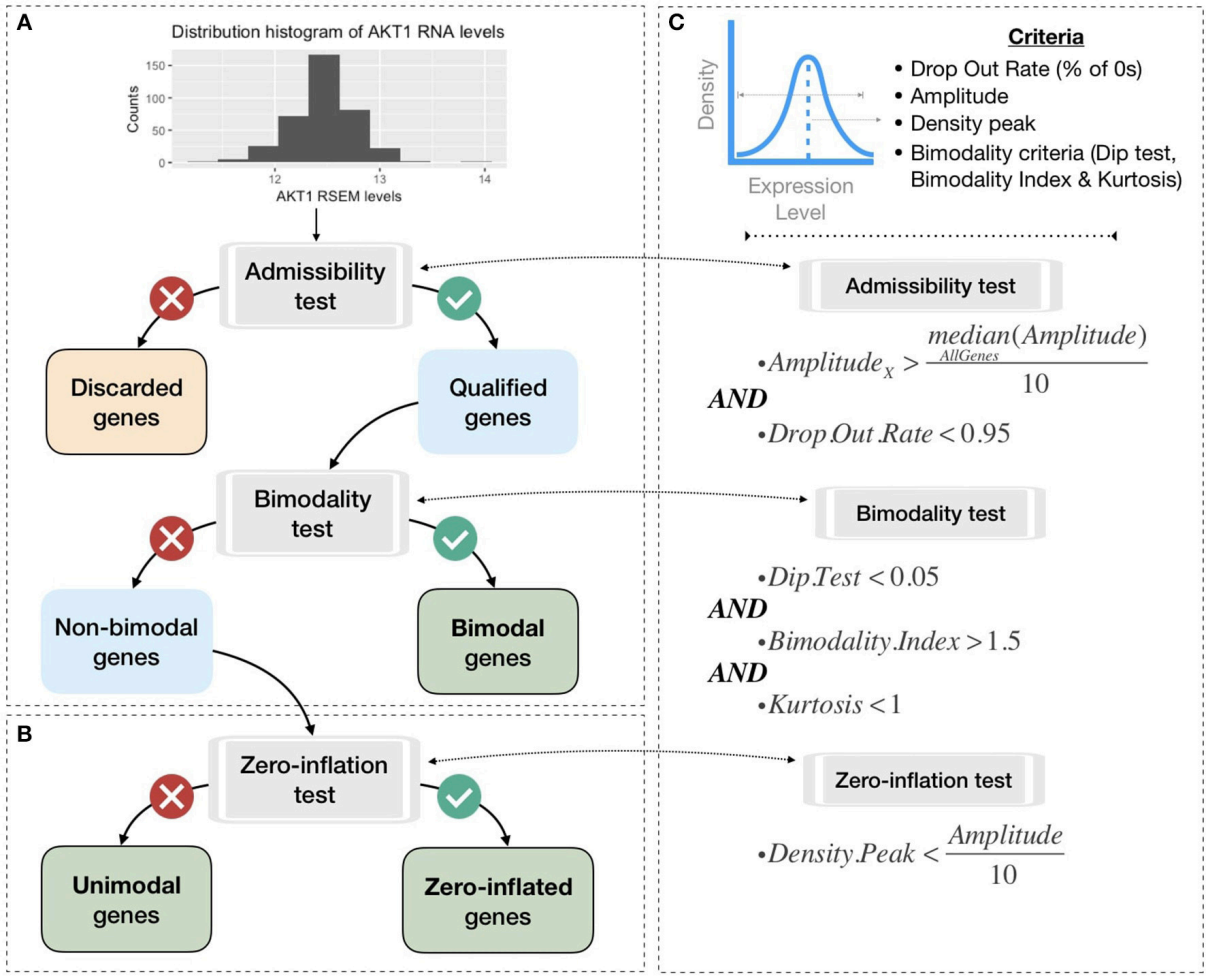
Processing pipeline to classify genes in different categories based on their expression pattern across the cohort. **(A)** Tests to separate bimodal from non-bimodal genes before subsequent binarization. **(B)** Test to classify non-bimodal genes in unimodal or zero-inflated genes. **(C)** Statistical and logical content of the various tests used in **(A,B)**, thresholds have been taken from the papers presenting each tool and are more precisely justified in the Methods section.

To be integrated into the logical model, continuous data must be either binarized or normalized between 0 and 1. To do so, gene expression data are first classified in three broad categories according to their distribution across samples: bimodal, unimodal, and zero-inflated distribution. Genes with different distributions are treated differently as summarized in Figure 3. Binarization and normalization methods different from the ones proposed here (e.g., Müssel et al., 2015; Jung et al., 2017) may also be used and directly integrated in the pipeline presented in the 2.4 section.

##### 2.3.2.1. Distribution classification

Non-variant genes are discarded based on the admissibility test: the test verifies that the gene expression is included in a sufficient range of values compared to other genes (i.e., a gene's amplitude across the cohort above one tenth of median amplitude across all genes) and contains a sufficient number of non-zero values (i.e., at least 5% of non-zero values). In single-cell transcriptomics terminology, the latter corresponds to a low drop-out rate.

In order to classify the remaining genes, we identify bimodal patterns based on three distinct criteria: Hartigan's dip test of unimodality, Bimodality Index (BI) and kurtosis.

The dip test measures multi-modality in a sample using the maximum difference between empirical distribution and the best unimodal distribution, i.e., the one that minimizes this maximum difference (Hartigan and Hartigan, 1985). Values below 0.05 indicate a significant multi-modality. In PROFILE, this dip statistic is computed using the R package diptest.

The Bimodality Index (BI) evaluates the ability to fit two distinct Gaussian components with equal variance (Wang et al., 2009). Once the best 2-Gaussian fit is determined, along with the respective means μ_1_ and μ_2_ and common variance σ, the standardized distance δ between the two populations is given by

(1) $$\begin{array}{r} \delta =\frac{|\mu_{1}-\mu_{2}|}{\sigma } \end{array}$$

and the BI is defined by

(2) $$\begin{array}{r} BI={\left[\pi \left(1-\pi \right)\right]}^{\frac{1}{2}}\delta \end{array}$$

where π is the proportion of observations in the first component. In PROFILE, BI is computed using the R package mclust.

Finally, the kurtosis method corresponds to a descriptor of the shape of the distribution, of its tailedness, or non-Gaussianity. A negative kurtosis distribution, especially, defines platykurtic (flattened) distributions, and potentially bimodal distributions. It has been proposed as a tool to identify small outliers subgroups or major subdivisions (Teschendorff et al., 2006). In our case, we focus on negative kurtosis distributions to rule out non-relevant bimodal distributions composed of a major mode and a very small outliers' group or a single outlier (an example of which can be seen in Figure S7).

Although dip test, BI and negative kurtosis criteria emerge as similar tools in the sense that they select genes whose values can be clustered in two distinct groups of comparable size, we choose to combine them in order to correct their respective limits and increase the robustness of our method (see bimodality test in Figure 3C). For that, we consider that all three conditions (Dip test, Bimodality Index and kurtosis) must be fulfilled in order for a gene to be considered as bimodal.

The thresholds of each test are inspired by those advocated in the papers presenting the tools individually. Dip test is a statistical test to which the classical 0.05 threshold has been chosen. In the article describing BI, authors explored a cut-off range between 1.1 and 1.5 and we chose 1.5 for the present work. Regarding kurtosis, the usual cut-off is 0, but since this criterion does not directly target bimodality, this criterion has been relaxed to *K* < 1. Several examples of the relative differences and complementarities between these criteria can be seen in Figure S7.

This method is enough to binarize continuous data as can be seen in Figure S8. However, to normalize continuous data, we need to further classify non-bimodal gene distributions among unimodal or zero-inflated, looking at the position of the distribution density peak. Then, based on this three-category classification of genes, we performed binarization and normalization processing as summarized in Figure S8.

Because the normalization of continuous data preserves more original information than its binarization, we will detail here only the normalization process. However, it should be noted that the preliminary classification of gene distributions into three distinct categories allows for a simple binarization (Figure S8).

Normalization functions are thus defined as follows:

$$\begin{array}{l} Bin:OriginalValues\to BinarizedValues\\ \;X\mapsto Bin(X)\\ Norm:OriginalValues\to NormalizedValues\\ \;X\mapsto Norm(X) \end{array}$$

##### 2.3.2.2. Bimodal genes processing: Gaussian mixture models

**Figure 4:**
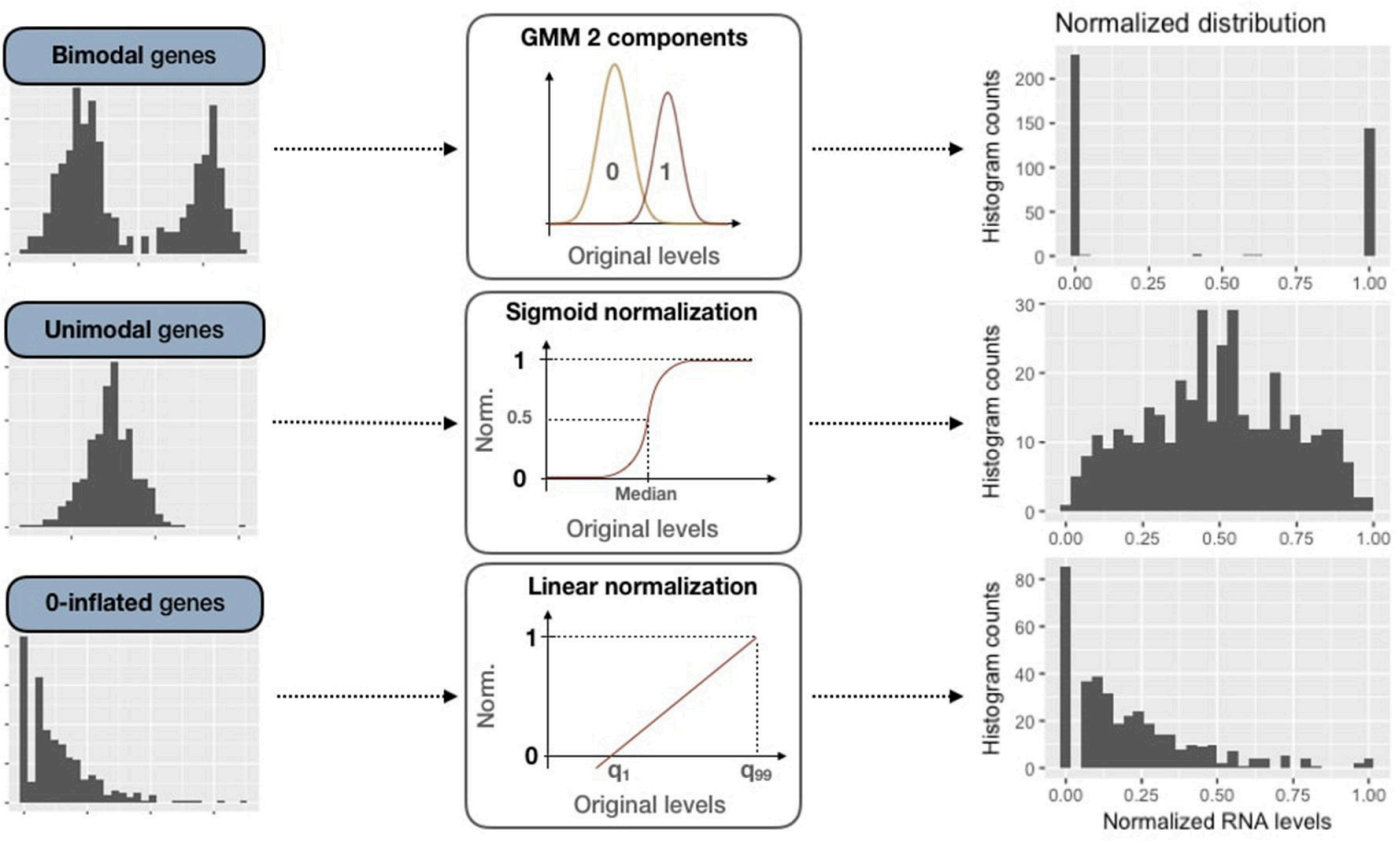
Normalization methods for expression data of genes of all three categories (bimodal, unimodal and zero-inflated). First column panels show examples of original patterns from the three categories. Second column panels illustrate the processing methods used for normalization (GMM 2-component, sigmoid normalization and linear normalization). Third column panels correspond to normalized distributions.

In PROFILE, a 2-component Gaussian mixture model is fitted using mclust R package resulting in a lower mode *M*_0_ and a upper mode *M*_1_ (Figure 4). Each data point *X* has a probability to belong to *M*_0_ or *M*_1_ such as

(3) $$\begin{array}{r} Prob\left(X_{gene_{i},sample_{j}}\in M_{0,gene_{i}}\right)+Prob\left(X_{gene_{i},sample_{j}}\in M_{1,gene_{i}}\right)=1 \end{array}$$

For these bimodal genes, the normalization processing is defined as:

(4) $$\begin{array}{r} Norm\left(X_{gene_{i},sample_{j}}\right)=Prob\left(X_{gene_{i},sample_{j}}\in M_{1,gene_{i}}\right) \end{array}$$

##### 2.3.2.3. Unimodal gene sigmoid normalization

For unimodal distributions, we transform data through a sigmoid function in order to maintain the most common pattern which is unimodal and nearly-symmetric. First of all, expression data are centered around the median, which is more robust than using the mean regarding outliers:

(5) $$\begin{array}{r} X_{gene_{i},sample_{j}}^{\prime }=X_{gene_{i},sample_{j}}-median_{gene_{i}}\left(X\right) \end{array}$$

Then data are normalized through the sigmoid function:

(6) $$\begin{array}{r} Norm\left(X_{gene_{i},sample_{j}}^{\prime }\right)=\frac{1}{1+e^{-\lambda X_{gene_{i},sample_{j}}^{\prime }}} \end{array}$$

Since the slope of the function depends on λ, we adapt λ to the dispersion of initial data in order to maintain a significant dispersion in [0, 1] interval: more dispersed unimodal distributions are mapped with a gentle slope, peaked distributions with a steep one. We map the median absolute deviation (MAD) on both sides of the median respectively to 0.25 and 0.75 to ensure a minimal dispersion of the mapping. First, the MAD is defined as:

(7) $$\begin{array}{r} MAD_{gene_{i}}\left(X\right)=median\left(|x_{i}-median_{gene_{i}}\left(X_{gene_{i},sample_{j}}\right)|\right) \end{array}$$

Therefore, to fulfill the proposed mapping, we solve:

(8) $$\begin{array}{r} \frac{1}{1+e^{\pm \lambda MAD}}=\frac{1}{2}\mp \frac{1}{4}, \end{array}$$

and derive:

(9) $$\begin{array}{r} \lambda =\frac{log_{e}\left(3\right)}{MAD} \end{array}$$

Thus, we obtain data normalized in [0, 1] for unimodal genes, as in Figure 4.

##### 2.3.2.4. Zero-inflated genes sigmoid normalization

Zero-inflated genes are characterized by a distribution density peak (computed in PROFILE with the density function of stats R package) close to 0 (Figure 3B). For this case, we linearly transform the initial distribution in order to maintain the asymmetric original pattern:

(10) $$\begin{array}{r} Norm\left(X_{gene_{i},sample_{j}}\right)=\frac{X_{gene_{i},sample_{j}}-min_{gene_{i}}\left(X\right)}{max_{gene_{i}}\left(X\right)-min_{gene_{i}}\left(X\right)} \end{array}$$

The transformation is applied to data between 1*st* and 99*th* quantiles to be more robust to outliers. Values below *q*_1_ or above *q*_99_ are respectively assigned to 0 and 1.

##### 2.3.2.5. Reference expression dataset

For the processing of expression data, two main options are available in PROFILE depending on what reference dataset is taken into account. We can either binarize/normalize genes based on distribution patterns across the whole cancer cohort or based on healthy patients. In the latter case, the type of gene distributions (bimodal, unimodal and zero-inflated) and the corresponding parameters (like inter-quartile range) are defined based on distribution patterns for healthy samples only, and the binarization/normalization is then applied on cancer patients. In the datasets under consideration in the present work, only the TCGA RNA dataset includes healthy samples. Except otherwise stated, genes are processed based on cancer cohort and not based on healthy samples.

### 2.4. Personalization of Logical Models Using Patient Data

**Figure 5:**
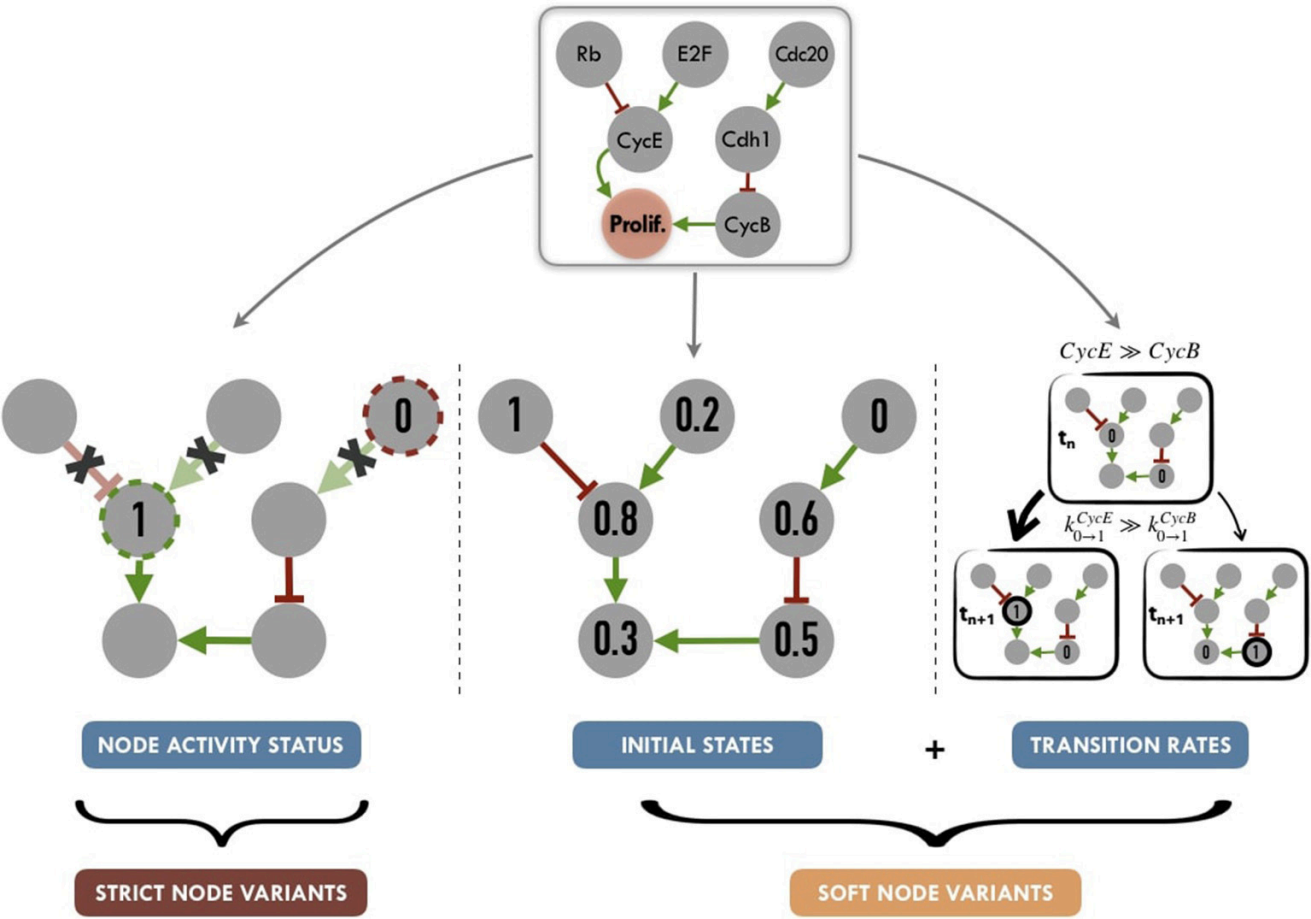
Graphical summary of personalization methods for logical models: node activity status (nodes set to a given value for the full run of the simulation, without consideration for logical rules), initial states (nods initialized to a given proportion of 0 or 1 states) and transition rates (weighted stochastic transitions depending on the assumed activity level of nodes). Tuning node activity status, we define *Strict Node Variants* or Strict NV, i.e., forcing the activity (either present or absent) of a node. Tuning both initial states and transition rates, we define *Soft Node Variants* or Soft NV, *i.e*, nodes whose initial activation proportion is close to that of the patient and whose transition rates promote maintenance in its state of activity.

Personalization has been defined here as the specification of a logical model with data from a given patient: each patient has a personalized model tailored to his/her data, so that all personalized models are different specifications of the same logical model, using data from different patients (Figure 1). Based on MaBoSS formalism and the processed patient data, there are several possibilities to personalize a generic logical model with patient data as represented in Figure 5.

#### 2.4.1. Activity of Model Nodes

One possibility to have patient-specific models is to force the value of the variables corresponding to the altered genes, i.e., constraining some model nodes to an inactive (0) or active (1) state. In order to constrain a node to 0 (resp. 1), the initial value of the node is set to 0 (resp. 1) and *k*_*up*_ (resp. *k*_*down*_) to 0 to force the node to maintain its defined state. For instance, the effect of a p53 inactivating mutation can be modeled by setting the node TP53 in the model and its initial condition to 0 and ignoring the logical rule of TP53 variable. These modifications are referred to as node activity in the logical model. This constraint affects the simulation trajectories and consequently may shift the trajectories in the solution state space (referred to as the state transition graph, STG) leading to a change in probabilities of the resulting stable states (very often, these nodes are the ones representing biological phenotypes that are used as read-outs of the model) (Grieco et al., 2013; Remy et al., 2015).

#### 2.4.2. Initial Conditions

Another possible strategy is to modify the initial conditions of the variables of the altered genes according to the results of the binarization/normalization. These initial conditions can capture different environmental and genetic conditions. Nevertheless, in the course of the simulation, these variables will be prone to be updated depending on their logical rules. These initial conditions can either be binary or continuous between 0 and 1, so both binarized and normalized profiles can be used. In the present study, we have only considered patients' expression data to be included as initial conditions, but PROFILE allows for more data types to be used as initial conditions.

#### 2.4.3. Transition Rates

Finally, as MaBoSS uses Gillespie algorithm to explore the STG, data can be mapped to the transition rates of this algorithm. In the simplest case, all transition rates of the model are set to 1, meaning that all possible transitions are equally probable. Alternatively, it is possible to separate the speed of processes by setting the transition rates to different values to account for what is known about the reactions: more probable reactions will have a larger transition rate than less probable reactions (Stoll et al., 2012). For this, different orders of magnitude for these values can be used. They are set according to the activation status of the node (derived from normalized or binarized values) and an “amplification factor,” designed to generate a higher relative difference in the transition rates, as follows:

(11) $$\begin{array}{r} k_{gene_{i},sample_{j}}^{up}=AmplificationFactor^{2\left(Norm\left(X_{gene_{i},sample_{j}}\right)-0.5\right)} \end{array}$$

(12) $$\begin{array}{r} k_{gene_{i},sample_{j}}^{down}=\frac{1}{k_{gene_{i},sample_{j}}^{up}} \end{array}$$

Thus, if a gene has a value of 1 based on its RNA profile, its transition rate from 0 to 1 (resp. from 1 to 0) will be 10^2^ (resp. 10^−2^) with an amplification factor of 100.

Note that in the present study, we have only considered normalized patients' expression data to be included as transition rates (RNA for METABRIC data and RNA or Protein for TCGA data). The influence of the amplification factor on the results is discussed in Section 1.6.2 and Figure S10 (Supplementary Material). Based on this analysis, we chose an amplification factor of 100.

#### 2.4.4. Synthetic Definition of Logical Model Personalization

We propose to summarize personalization methods in two different strategies (Figure 5). One one hand, applying *Strict Node Variants* (Strict NV) method, nodes for which data are available, are set to a given value for the whole simulation. For these nodes, logical rules are no longer in use, as they will always have a given value (0 or 1).

On the other hand, combining *Initial States* and *Transition Rates* modifications, we define a *Soft Node Variants* (Soft NV) method. Using this method, if a given node has a normalized value of 0.8 after data processing (based on proteins levels for instance), it will be initialized as 1 in 80% of the stochastic trajectories, its transition rate *k*_0 → 1_ will be increased (favoring its activation) and its transition rate *k*_1 → 0_ will be decreased (hampering its inactivation). These changes increase the probability that this node will remain in an activated state close to the one inferred from the patient's data, while maintaining the validity of its logical rule. Thus, Soft NV appears as a smoother way to shape logical models' simulations based on patient data.

#### 2.4.5. Combinations of Data Types

The choice of which data types to include and where to map these data on the modeling framework is dependent on the goals of the study. If mutations, CNA and gene and protein expression data are provided for a given patient, one could include all these data types as follows: nodes corresponding to mutations and CNA could be used to specify model nodes (set to 0 or 1 if they are inhibiting or activating mutations or if they are homozygous deletions or amplifications), and transition rates could be modified to account for gene and protein expression levels.

Mapping different data types with different personalization methods avoids potential conflicts. However, combining different data types with the same personalization method raises some ambiguity issues. For instance, a gene can be inferred as a loss of function from the mutation data and can be found as amplified from CNA data. In this case, we consider that the information from mutations always overrides the information coming from CNA or binarized RNA/protein. Since both RNA and protein expression are available in the TCGA dataset, we explored the possibility to combine the two data types as follows: the RNA expression level is taken into account to define soft node variants only if there is no corresponding data in the protein dataset for that specific node. In the section 3.2, we present different choices that can be made according to the studied goals and data availability and in section 3.3, we analyze which combination is best suited to explain our patients' clinical data.

### 2.5. Comparison With Clinical Data

In order to assess the relevance of the different scenarios of model personalization, we investigate the correlation with biological and clinical factors.

For METABRIC dataset, signatures from the Molecular Signature Database (MSigDB) described in Liberzon et al. (2015) were used to classify the relevance of *Proliferation* and *Apoptosis* probabilities obtained from different personalization methods. We selected the Hallmarks “G2M Checkpoint” (resp. “Apoptosis”), a gene set composed of 200 genes (resp. 161) to correlate with the *Proliferation* (resp. *Apoptosis*) model probabilities. Genes used to personalize the models are excluded from the gene set, which reduces it to 185 (resp. 150) genes. Signature scores are then computed with the Gene Set Variation Analysis (GSVA) method, described in Hänzelmann et al. (2013) and implemented in GSVA R package. Correlations are assessed based on Spearman rank method and 95% confidence intervals are obtained by bootstrap (*n* = 1, 000). For the METABRIC cohort, the patient's Nottingham prognostic index (NPI) and survival data are also gathered. NPI is a prognostic score based on clinical features such as tumor size, tumor grade and node status.

Regarding the survival data, there is data for all but one of the 1980 METABRIC patients. The overall survival time points are between 0 and 355 months with a median survival time of 283 months and 646 events (patients died of disease). Kaplan-Meier fits are obtained using the survival R package.

### 2.6. Availability

All the scripts and models are freely available on GitHub (https://github.com/sysbio-curie/PROFILE) and are distributed open source under the BSD 3-clause license. This repository can be referred to with its own DOI: (https://doi.org/10.5281/zenodo.1491229).

## 3. Results

### 3.1. Breast Cancer Data Processing

Our framework has been applied to 2,509 breast cancer patients' molecular data that were collected from METABRIC. Patients' data types include exome mutations, CNA and RNA expression as well as clinical data such as survival data. One thousand nine hundred and four patients of the 2,509 total have all these data types available (Curtis et al., 2012; Pereira et al., 2016) (Figure S8A). Data were processed as described in previous sections.

The logical model of cancer pathways (Fumiã and Martins, 2013) was chosen as a working example as it is a generic model with a relatively big number of nodes that span several pathways relevant to cancer. This model was initally used to study the effects of microenvironment conditions, to simulate the response to driver mutations in colorectal cancer progression and the effect of genes' perturbations as therapeutic targets (Fumiã and Martins, 2013).

Data from the METABRIC dataset that were relevant to the model were selected. Focusing on the 110 genes overlapping with nodes of the logical model, exome sequencing resulted in 2,659 mutations, of which 1,431 mutations were inferred as loss of function and 1,228 as gain of function. Besides, 634 mutations have unknown or silent effects and therefore were not considered. These 3,293 model-related mutations represent 19% of all mutations of the METABRIC dataset. Note that these numbers show the intersection of a generic model and a breast-cancer-specific dataset, so this percentage could be further increased by using a model with breast-specific pathways. Patients' profiles were found to have up to 7 mutations with most patients having only one assigned mutation. PIK3CA and TP53 were found to be the most frequently mutated genes.

For CNA data, patients' profiles had up to 19 perturbations, with a median number of 2. MYC gene was the most frequent gene with copy number alterations.

RNA expression data were processed and genes were separated in bimodal, unimodal and zero-inflated categories (Figure 3 and section 2.3). All model-related genes in METABRIC cohort were found to be unimodal. Note that bimodal genes occur in several biologically meaningful situations like fusion genes such as ERG in prostate cancer or hormone genes such as ESR1 in breast cancer. We chose to explore the results of the METABRIC data with a model built specifically for breast cancer analysis (Zañudo et al., 2017) in order to assess the importance of including cancer-specific genes. Indeed, ESR1 is present in the breast-specific logical model analyzed in Supplementary Material.

The methods of binarization and normalization are applied to each data type according to the previously presented rules (Figure 4 and Figure S8).

We further compared our binarization method to an existing tool, RefBool framework (Jung et al., 2017), using the same METABRIC dataset. This tool uses a set of reference distributions and it results in *p*-values for each sample and gene, assessing the significance of its putative binarization. Using 0.05 as a binarization threshold for RefBool *p*-values on the whole METABRIC RNA dataset (1904 samples and 24368 genes), around 4.4 million values were binarized (9.5% of the total). All of these binarizations resulted in active nodes and thus set to 1. Notably, RefBool was designed to use a reference dataset to binarize new data. Due to the lack of a reference healthy dataset in METABRIC, the whole dataset has been used as its own reference: each gene was compared to the distribution of that gene across all samples. Comparatively, our method results in 2.8 million of binarized values (6.1% of the total), respectively 4.2% of 1 s and 1.9% of 0 s. There seems to be a trend for RefBool in METABRIC dataset to emphasize positive outliers at the expense of negative ones, even for roughly symmetric unimodal distributions (Figure S18). Some examples of this dataset can be studied in Supplementary Material, together with the analysis on TCGA dataset, that bears healthy samples, and should be a better showcase to RefBool capabilities (Figure S19).

### 3.2. Personalization of a Generic Logical Cancer Model With Breast Cancer Data

**Figure 6:**
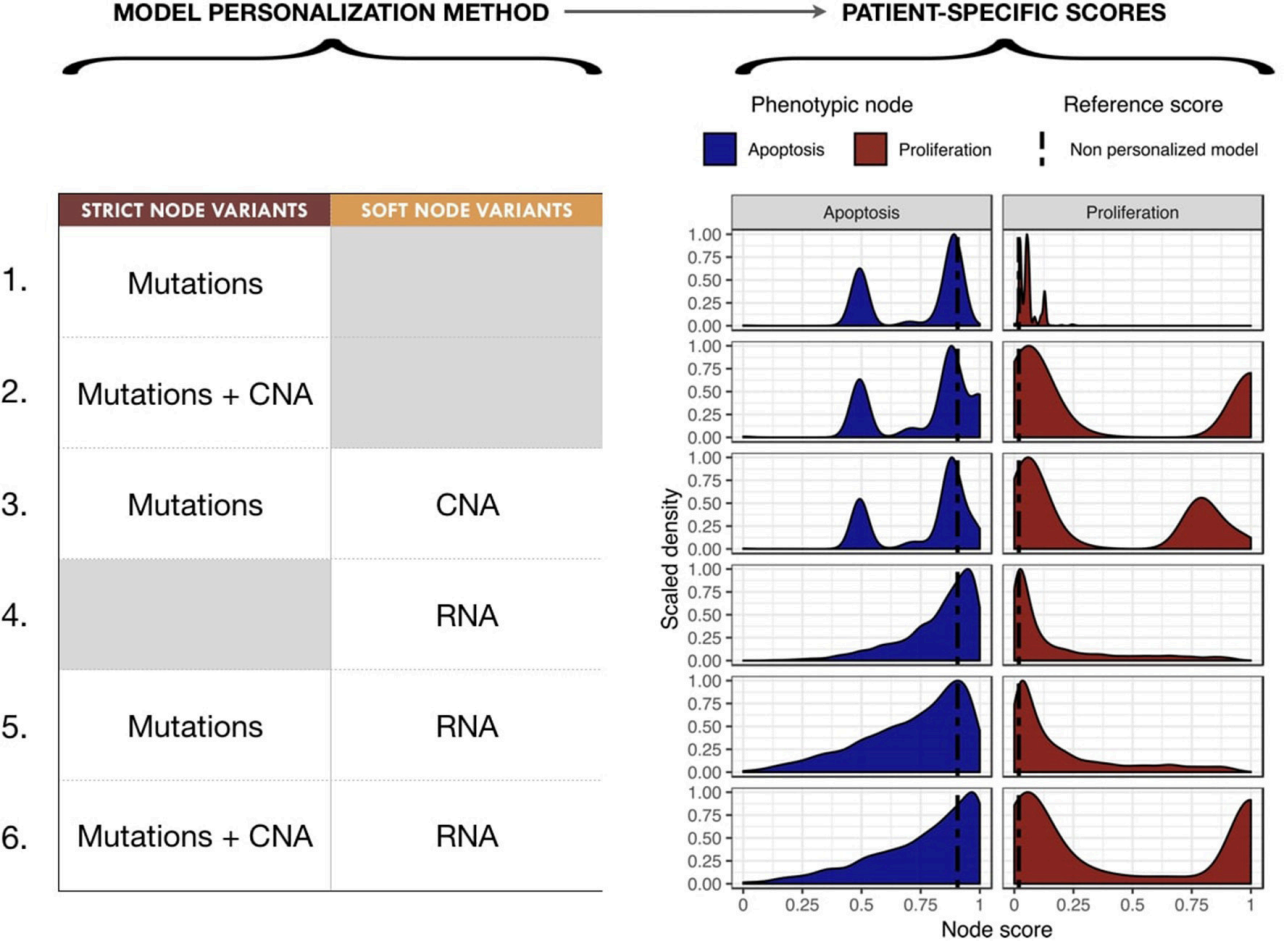
Impact of different model personalization methods on the distribution of phenotypic nodes *Proliferation* and *Apoptosis*, using a generic cancer model and METABRIC data. On the left, description of data types used as *Strict* or *Soft Node Variants* to personalize the model resulting in different phenotypic probabilities' distributions across the cohort, as shown on the right. Dashed lines correspond to the probabilities of the phenotypes obtained from the original model without any personalization: 0.019 for *Proliferation* and 0.906 for *Apoptosis*.

We proceeded to personalize the logical model using different types of data and several data integration methods, such as on the activity of the nodes, the initial conditions and the transition rates. The effect of integrating different data at different levels of the model are represented by different phenotypes' distributions that can be used to study the respective effects of model personalization methods in Figure 6. Note that the probabilities for the wild type conditions are 0.019 for *Proliferation* and 0.906 for *Apoptosis* and are represented as a black dashed vertical line in Figure 6.

Using mutation data as a forced activity (either present or absent) of a node of the model (termed *Strict Node Variants* or Strict NV throughout the text), resulted in the distribution of *Proliferation* probabilities around the value 0.05 and the distribution of *Apoptosis* probabilities around two values (0.5 and 0.85) in Figure 6 (upper panels, case 1). It is important to note that as these data are discrete and sparse, this causes the *Proliferation* distribution to be quite sharp. The distribution becomes smoother when exome mutations and CNA are both considered as Strict NV of the model and peaked around two values (0.05 and 1 for *Proliferation* and 0.5 and 0.85 for *Apoptosis*), as shown on Figure 6, case 2. Using CNA information as *Soft Node Variants* (Soft NV) and mutation as Strict NV, the highly proliferative mode is slightly decreased, consistent with less stringent constraints (Figure 6, case 3). When only RNA expression levels are used as modified transition rates, the resulting distribution of phenotypes' probabilities is more dispersed (Figure 6, case 4) and only one lowly proliferative peak appears. Adding mutations information as Strict NV does not shift the probabilities' distributions (case 5). Lastly, when we consider mutations and CNA as nodes' activity and RNA expression levels as modified transition rates, it results in a combination of the previously observed patterns (Figure 6, case 6).

Nevertheless, the generic logical model we use here does not take into account key genes in breast cancer progression such as hormone receptors and their associated signaling networks. As previously mentioned, a breast-cancer-specific model (Zañudo et al., 2017) was investigated using the same METABRIC dataset to personalize breast patient-specific models with similar trends to those of the generic model's study (Figure S12). Zañudo et al. (2017) model generates narrower distributions and therefore less discriminating probabilities from one patient to another, which is mainly due to the fact that it captures less information due to its lower number of nodes (especially with sparse data such as mutations). For these reasons, and having in mind the methodological scope of present work, we will focus on the discussion on results of the more comprehensive generic model.

In order to present the use of one model with more than one dataset, PROFILE method was also done and analyzed using TCGA molecular data on Fumiã and Martins (2013) generic model (Figure S14).

Figures such as Figure 6 are useful to identify the integration of which data in which part of the model has a greater impact in the change in phenotypes' distributions, but say little about the biological relevance of these distributions. To further investigate which combinations of methodology provides better biological or clinical insights, we compared these models' results to several signatures or clinical factors used in breast cancer studies.

### 3.3. Selecting Personalization Methods Using Correlation of Phenotypic Probabilities to Signature Scores

To classify the relevance of the six personalization methods presented in the previous section, we studied the correlations of the probabilities of the model phenotypes with representative signatures of the same phenotypic processes. This methodology allows to classify the different personalization methods and to study which one is better suited to describe the diversity of patients when tailoring a given model to a given dataset.

**Figure 7:**
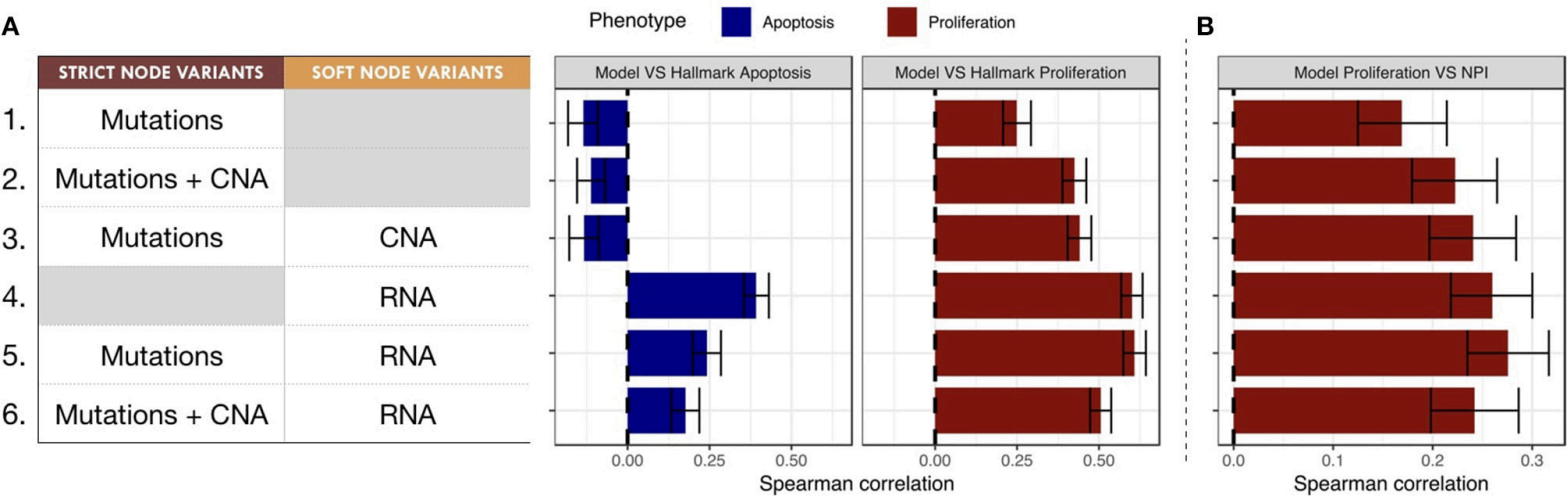
Biological and clinical classification of phenotypic probabilities from personalized models. **(A)** Model personalization methods used and the corresponding Spearman rank correlation between phenotypic probabilities from personalized model and the corresponding Hallmark signatures score based on RNA gene sets. **(B)** Spearman rank correlation between personalized *Proliferation* probabilities and the Nottingham Prognostic Index (NPI) score based on clinical features (size and grade of tumor, node status).

The Spearman rank correlations of the *Apoptosis* probabilities from personalized models with the RNA-based "Apoptosis" signature defined in the Hallmarks (Liberzon et al., 2015) gene set was computed (Figure 7A). Sparse binary data (when using mutations or CNA data) appear to be a poor choice to recover a consistent Apoptosis probability with the logical models (cases 1, 2 and 3). Only models personalized with RNA data as Soft NV are able to mimic an Apoptosis behavior consistent with the signature.

When comparing the *Proliferation* probabilities from the models to the Hallmarks' "G2M Checkpoint" signature (Figure 7A), personalized models are able to capture consistent behavior regardless of the type of data used as input. Nevertheless, the best Spearman rank correlations coefficients used as classifiers singled out the cases that use RNA as Soft NV (cases 4, 5 and 6), specially when the activity of nodes was fixed by mutations and transition rates by RNA values (case 5, mean Spearman's ρ of 0.61). In spite of their smaller correlation, the first three cases are also of interest since they only make use of originally sparse and discrete information: mutations and CNA data used as Strict and/or Soft NV. For instance, in case 3, using mutations as Strict NV and CNA as Soft NV, personalized models are able to retrieve 44% of proliferation information contained in RNA-based “G2M Checkpoint” signature (Figure 7A, case 3).

Similarly, when comparing the probabilities of the *Proliferation* phenotype to NPI scores (Figure 7B), a purely clinical index that is not based on omics data, we observe the same trends for correlations, but with decreased coefficients. This supports the potential of these personalized models to partially identify clinical information as discussed in the survival data in section 3.5.

### 3.4. Clinical Subgrouping of Patients' Specific Model Outputs

**Figure 8:**
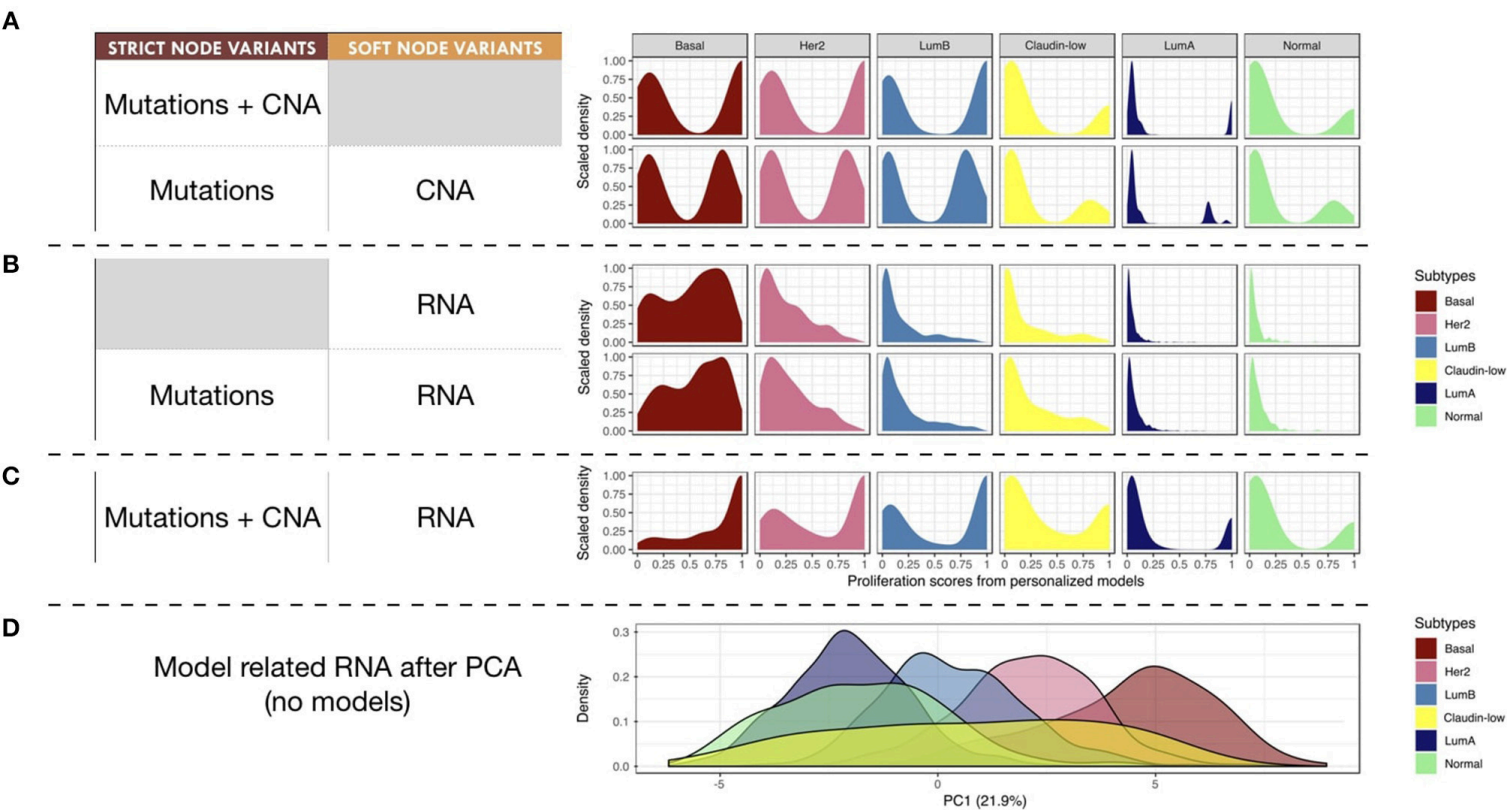
Breast-cancer subtypes and personalized logical models. **(A–C)** Patterns of *Proliferation* probabilities per subtype with different personalization methods results in different grouping of subtypes. **(D)** Densities of breast-cancer subtypes along first principal component, PCA transformation based on METABRIC RNA data limited to the 114 genes described in the logical model ; percentage of variance explained by PC1 in parenthesis.

Next, we studied the relationship of our patients' specific model probabilities to the PAM50 subgrouping, defined by the expression of 50 genes (Parker et al., 2009). For this, *Proliferation* probabilities from the personalized models were compared across subtypes (Figures 8A–C).

Using only mutations and CNA (Figure 8A), two different patterns may be observed: Basal, Her2 and Luminal B patients have balanced *Proliferation* bimodal probabilities with both lowly and highly proliferative patients. The second pattern involves Claudin-low, Luminal A and Normal-like patients that are mainly lowly proliferative with a smaller highly-proliferative mode. This grouping of subtypes, based on distribution trends, is consistent with the distinct proliferative behaviors of breast-cancer subtypes as described in Prat and Perou (2011): although similar in some aspects Luminal subtypes are distinguished by the more proliferative aspect of Luminal B; Basal and Her2 subtypes are also considered as aggressive tumors in contrast to Luminal A and Normal-like; Claudin-low subtypes have mixed behaviors depending on conditions but are usually described as lowly proliferative *in vivo*. The trends captured by the model are therefore consistent with clinical knowledge.

When personalizing logical models with RNA but no CNA (Figure 8B), only the proliferative nature of the Basal subtype seems to be well described, even when using mutation data. When combining RNA and CNA data (Figure 8C), the previously described clinical trends are again observed with clearer distinctions between subtypes.

In order to provide a reference of subtyping using omics data, a Principal Component Analysis (PCA) was performed taking into account the RNA expression levels of the 114 genes related to all nodes of the model (Figure 8D). The first principal component (PC1) of this PCA captured the different molecular subtypes and sequentially separated different subtypes (Luminal A, Luminal B, Her2 and Basal). This analysis shows a smoother and more linear distribution of the different subtypes, while personalized models seem to assign them more discrete patterns.

### 3.5. Survival Analyses of Patients' Specific Model Outputs

As a follow-up to the correlation studies of phenotypes' probabilities and clinical NPI scores, METABRIC survival data were correlated to the *Proliferation* and *Apoptosis* probabilities. For the survival analysis, thresholds needed to be set for the probabilities for each phenotype in order to separate between two groups: high and low. These thresholds were defined using the median for each phenotype probability across the cohort. Thus, each patient was grouped into two groups (high or low) for each phenotype (*Proliferation* or *Apoptosis*).

**Figure 9:**
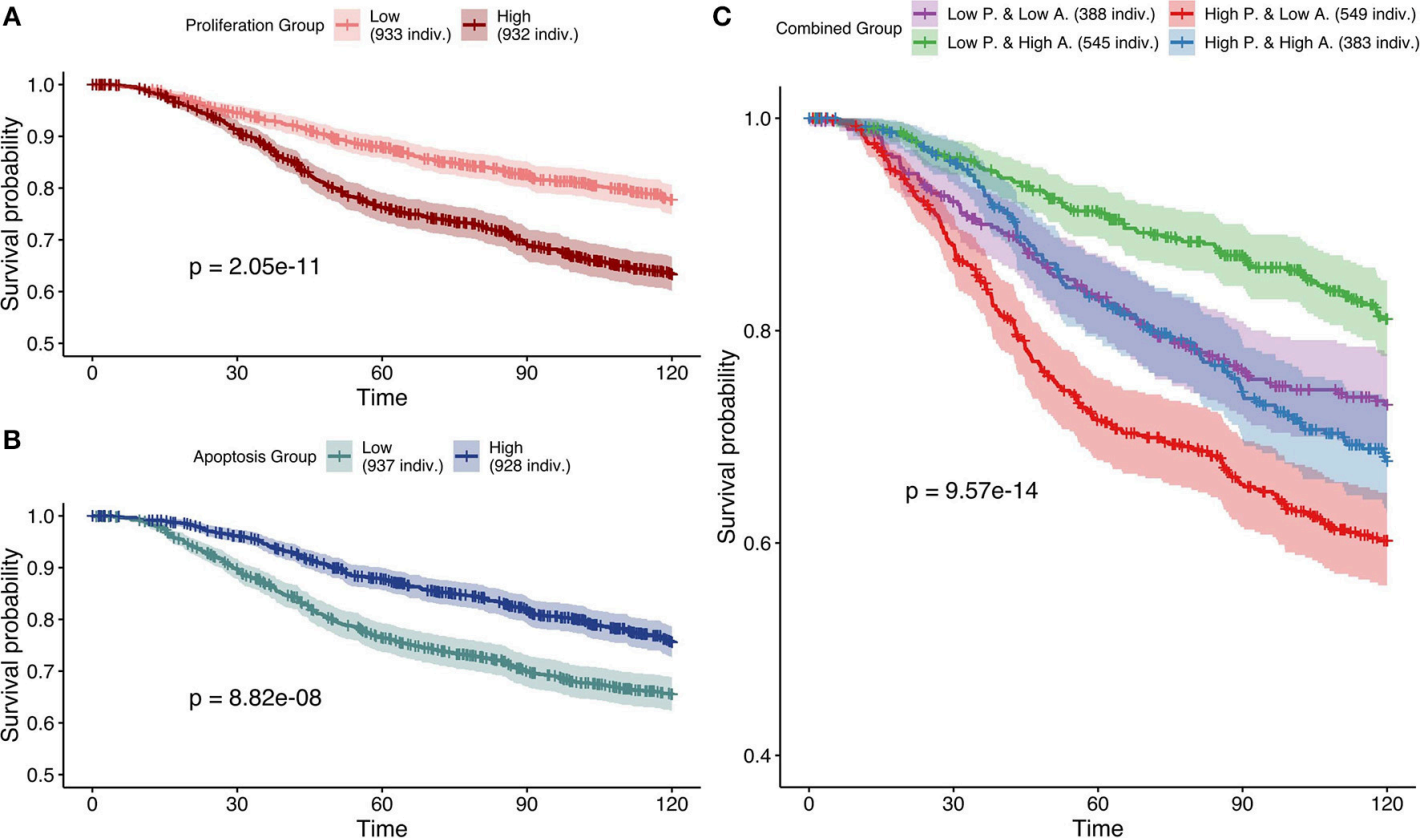
Survival analyses of METABRIC samples from which exome mutations are used as *Strict Node Variants* and CNA as *Soft Node Variants* in the model (case 3). All *p*-values are derived from a log-rank test. **(A)** Survival curves with high and low *Proliferation* groups. **(B)** Survival curves with high and low *Apoptosis* groups. **(C)** Survival curves with combined groups.

Studying simulation results from case 3 (mutations as Strict and CNA as Soft NV), thresholds of 0.12 and 0.87 were determined for *Proliferation* and *Apoptosis* phenotypes respectively. Kaplan—Meier plot (Kaplan and Meier, 1958) for *Proliferation* low and high probabilities' groups were significantly different (log-rank test, *p* = 2.05*e*^−11^) and low proliferative patients' models had better prognostic than the high ones (Figure 9A). When considered as a continuous biomarker, *Proliferation* appeared significant in a Cox model with a *p*-value of *p* = 2.13*e*^−8^.

Similarly, Kaplan—Meier plot for *Apoptosis* low and high probabilities' groups were significantly different (log-rank test, *p* = 8.82*e*^−8^) and high apoptotic patients' models had better prognostic than the low ones (Figure 9B). When considered as a continuous biomarker, *Apoptosis* appeared significant in a Cox model with a *p*-value of 1.09*e*^−8^. The observation of survival curves for high apoptotic or low proliferative patients' models having a much better prognostic than the opposite phenotypes (Figures 9A,B) is in accordance with the underlying cancer biology and is an implicit validation on the relevance of the model and its simulations.

We next combined both thresholds to separate patients in four groups (high and low *Proliferation* and high and low *Apoptosis*) (Figure 9C) that was also significantly different (log-rank test, *p*-value of 9.57*e*^−14^). Using this combination, the best prognosis was for patients' models with low *Proliferation* and high *Apoptosis* and the worst prognosis was associated to patients' models with high *Proliferation* and low *Apoptosis*. Groups with the other labels (either high *Proliferation* and high *Apoptosis* or low *Proliferation* and low *Apoptosis*) had mild prognoses. This observed behavior is fully consistent with the expected influence of proliferation and apoptosis in cancer prognosis. Thus, using sparse and binary data, we show that personalized logical models result in a meaningful stratification of patients.

**Figure 10:**
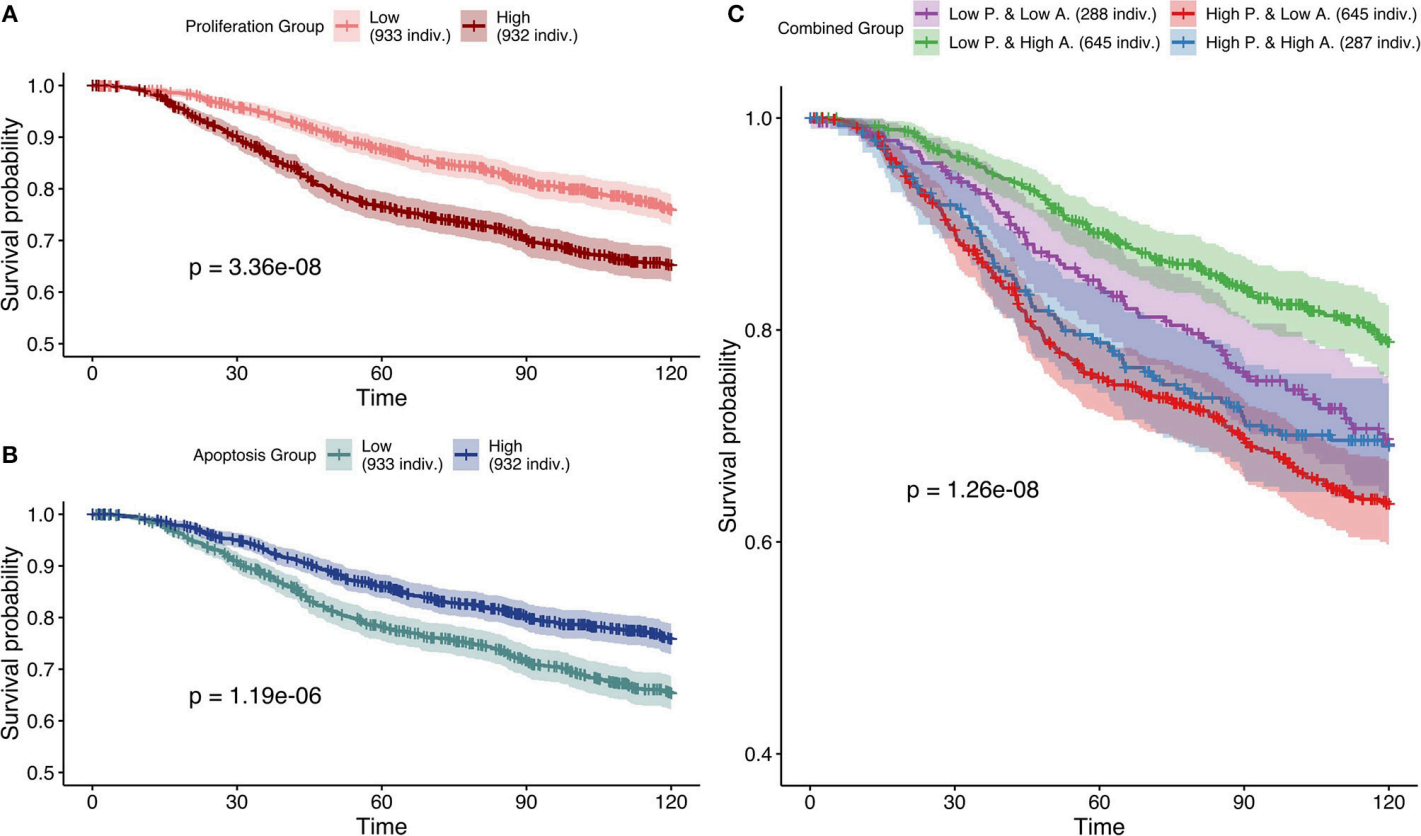
Survival analyses of METABRIC samples from which exome mutations are used as *Strict Node Variants* and RNA as *Soft Node Variants* in the model (case 5). All *p*-values are derived from a log-rank test. **(A)** Survival curves with high and low *Proliferation* groups. **(B)** Survival curves with high and low *Apoptosis* groups. **(C)** Survival curves with combined groups.

Next, based on Figure 7, the most effective personalization method was selected (case 5 using mutations as Strict and RNA as Soft NV) and its survival analysis had similarly consistent behaviors (Figure 10). Nevertheless, using only RNA as Soft NV (case 4 of Figure 7), *Proliferation* remains very significantly correlated with survival data but *Apoptosis* is not (Figure S16), supporting the importance of mutations data to retrieve biologically consistent behaviors.

Based on Figures 7–10 we conclude that for an optimal integration of the data available in this logical model, the best combinations are to binarize mutations and treat them as Strict NV, and to integrate RNA as Soft NV. Replacing RNA with CNA data results also in largely consistent behaviors with sparser data.

We conclude that our personalization protocol is useful to build data-tailored models that can capture patient-specific phenotypes' behaviors which correlate to survival data.

## 4. Discussion

In order to reach its full potential, personalized medicine needs precise mathematical models, and this will only be achieved with models tailored to the data for a given patient. These patient-specific models can be of great help to study patient-tailored drug combinations or the different drug responses in a group of patients with similar profiles and to advice the clinical oncologist as to the optimal treatment to choose for a given patient. The methodology presented here is a first step toward the personalization of a logical model to different patient profiles such that their results can be matched to clinical data and patients' subgrouping.

Our PROFILE framework is able to use different data types (mutation, CNA and gene and protein expression data) and incorporate them at different levels of the logical modeling formalism. The personalization strategies presented here have been compared to well-established signatures and NPI score, and the outcomes of these patient-specific models have shown to correlate well with clinical data. Any other relevant clinical measure could be used, especially more specific features corresponding to molecular mechanisms studied in the models. Notably, some choices on which data to include in the specification of the models are better than others when studying the correlation of the phenotypic probabilities of the logical model to signatures or the model ability to differentiate patients by prognostic outcome. To summarize, associating genetic mutations with the most stringent personalization method (i.e., Strict NV, constraining activity of nodes to either 0 or 1) and variation of copy number and expression levels with more permissive and stochastic personalization methods (i.e., Soft NV, intervening in initial states and transition rates) can be seen as biologically consistent. It is indeed expected that a genetic mutation can have a very strong and lasting effect that makes the gene independent of any regulation such as in the loss of function mutations. Conversely, the RNA expression level will affect the activity level of the genes but may not alter its regulation.

Using our PROFILE methodology, we are able to provide guidelines regarding the patient-data personalizations of logical models. Firstly, it is important to consider the nature of the node (gene or protein) in order to match the proper data type to the node. In the generic model used in our study, most of the nodes are supposed to be proteins, therefore it would be advisable to focus on protein data, which is unfortunately unavailable in the METABRIC dataset. In any case, the proposed framework could be easily adapted to the ideal case where each node would have a well-defined nature and a proper mapping of the corresponding data types. It is important to note that in the context of phospho-proteomic data (like RPPA's phosphosites), highly phosphorylated species can correspond to an inactive state that must be taken into consideration as mentioned in Supplementary Materials with TCGA data.

Secondly, healthy samples should be used if they are available in the dataset. Using an independent healthy samples for RNA normalization in TCGA dataset not only improved the correlation performances (Figure S15, case 4) but also the qualitative trend of the results (Figure S17). It can be seen that using healthy samples instead of cancer samples as a reference for RNA normalization results in a significant shift of the distribution toward high *Proliferation* model probabilities (Figure S17).

Thirdly, to improve the results of personalized logical models, the model used must be big enough, but also cover specificities of the cancer under study. Models should not be too generic, as they should include important read-outs of cancer types such as AR for prostate or ER and BRCA1 for breast cancer allowing them to better separate cancer subgroups. Also, they should include a sufficiently meaningful number of genes in order to be able to differentiate among patients.

In order to achieve clinically relevant models, it will be necessary to bring together the best of both worlds: large models able to integrate most alterations of common cancer pathways (e.g., DNA repair) and cancer-specific nodes (e.g., hormone receptors) able to explain the particular behavior of each cancer.

As perspectives, we plan to explore methods that will allow to use the solutions of the logical model for patient-specific studies. One possibility that would allow for personalized drug treatments is to integrate drug interactions in these personalized models, uncovering patient-specific drug targets whose behaviors might depend on environmental conditions. Another possibility that would enable a better patient stratification is to compute the Hamming distance of a binarized profile of a patient with each of the stable states obtained by the non-personalized model. That way, a patient can be considered "closer" to a given phenotype, such as *Proliferation, Apoptosis* or *Senescence*, etc. This approach raises problems such as how to treat attractors such as limit cycles, which are usually found in logical models, since this comparison can only be done on stable state solutions. We have started exploring this possibility (Cohen et al., 2015) and some work has been done by other groups in this direction (Dorier et al., 2016).

In conclusion, our PROFILE methodology allows to build precise mathematical models that captures the heterogeneity of patients profiles and their diverse behaviors. These logical models, which are properly specified with patient information, would enable clinicians to test personalized drugs combinations or therapeutic strategies *in silico* and pave the way to precision medicine.

## Author Contributions

LC and EB designed the project. JB, LC, PT, and AM participated in the conceptualization of the methodology. PT and JB designed the methods for the generation of patients' profiles from datasets. JB set up the pipeline for binarization and normalization of the data, generated patients' profiles from datasets, adapted these to the model and performed the personalization of models. PT and AM selected and analyzed the logical models. Manuscript was written by JB, AM, and LC and all authors read and edited it.

### Conflict of Interest Statement

The authors declare that the research was conducted in the absence of any commercial or financial relationships that could be construed as a potential conflict of interest.

## Footnotes

^1^http://www.cbioportal.org/index.do
