# Supplementary material for: Personalization of Logical Models With Multi-Omics Data Allows Clinical Stratification of Patients
Source: Front Physiol. 2019 Jan 24;9:1965. doi: 10.3389/fphys.2018.01965 (PMC6353844; doi:10.3389/fphys.2018.01965)
Supplement: Supplementary file 1 [file Image_1.pdf]

Supplementary Material: Personalization of Logical Models With Multi-Omics Data Allows Clinical Stratification of Patients

1 SUPPLEMENTARY METHODS

1.1 Logical modeling principles

Mathematical models serve as tools to answer biological questions in a formal way, to detect blind spots and thus better understand a system, to organize, into a consensual and compact manner, information dispersed in different articles, to identify new hypotheses and to test experimental hypotheses and predict their outcome (Montagud et al., 2017). The use of logical modeling to study biological problems has been used for some years and has delivered insights in the mechanism of cancerous cells, for exploring cell fate decisions, or particular dysfunctions in biological processes (Calzone et al., 2010; Rodríguez et al., 2012; Kazemzadeh et al., 2012; Schlatter et al., 2009; Ríos et al., 2015; Martinez-Sanchez et al., 2015). Logical models are particularly appropriate when the question is qualitative, or when the data we can gather are semi-quantitative (Abou-Jaoudé et al., 2016).

1.1.1 Glossary of terms used in logical modeling

Boolean - or discrete - models are based on the logical formalism, which relies on a regulatory graph and a list of logical rules associated to each of the nodes of the graph. There exists another graph, the state transition graph or STG, which recapitulates all the states of the nodes and the possible transitions from one state to another depending on the logical rules. The form of the graph will depend on the updating strategy chosen (either all nodes are updated at once or nodes are updated one at a time). The state transition graph informs on the existence of the two types of attractors of the model: stable steady states or limit cycles. In this study, we do not compute the state transition graph, because of the size of the networks we present, but rather perform stochastic simulations using Gillespie algorithm to explore the model solution space.

Below, we briefly introduce some terms related to logical formalism that are used throughout the manuscript. For a more thorough description, we invite the readers to explore more in-depth reviews such as:

- Saadatpour and Albert. Boolean modeling of biological regulatory networks: A methodology tutorial (Saadatpour and Albert, 2013).
- Abou-Jaoudé *et al*. Logical modeling and dynamical analysis of cellular networks (Abou-Jaoudé et al., 2016).
- Le Novère. Quantitative and logic modelling of molecular and gene networks (Le Novère, 2015).

Regulatory or influence network: The network is composed of nodes and edges, where nodes correspond to entities (e.g., genes, proteins, complexes, phenotypes or processes) and edges to influences, either positive or negative, which illustrate the possible interactions between two entities. Positive edges can represent the formation of active complexes, mediation of synthesis, catalysis, etc. and negative edges

inhibition of synthesis, degradation, inhibiting (de)phosphorylation, etc. Such regulatory networks are easily translatable to Boolean models. An example of an influence network can be found in Figure S5a).

We provide a small example of a toy model to illustrate the concepts presented here. The regulatory network is composed on 3 nodes: A, B and C. A activates B (green edge), B inhibits A (red edge) and C is an input activating A.

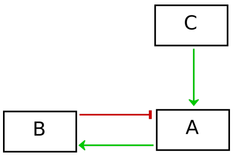

Figure S1: Regulatory network of a toy model of three genes: A, B and C.

Logical rules: Each node of the influence network has a corresponding Boolean variable associated to it. The variables can take two values: 0 for absent or inactive (OFF), and 1 for present or active (ON). These variables change their value according to a logical rule assigned to them. The state of a variable will thus depend on its logical rule, which is based on logical statements, *i.e.*, on a function of the node regulators linked with logical connectors AND, OR and NOT.

These operators can account for what is known about the biology behind these edges. If two input nodes are needed for the activation of the target node, they will be linked by an AND gate; to list different means of activation of a node, an OR gate will be used. For negative influences, a NOT gate will be utilized. Thus for each node, a logical rule is associated. The rules corresponding to the toy model (Figure S1) are the following:

```
A = !B & C
B = A
C = input
```

A is updated to 1 in the absence of B and the presence of C. B is activated if A is present. C is an input of the model.

State transition graph: In a Boolean framework, the variables associated to each node can take two values, either 0 or 1. We define a model state as a vector of all node states. All the possible transitions from any model state to another are dependent on the set of logical rules that define the model. These transitions can be viewed into a graph called a state transition graph, or STG, where nodes are model states and edges are the transitions from one model state to another. That way, trajectories from an initial condition to all the final states can be determined. One such transition can be seen in Figure S2 or S3.

The transition graph can contain up to 2^n model state nodes; thus, if n is too big, the construction and the visualization of the graph becomes resource consuming.

Asynchronous/synchronous updates: There are two strategies to construct this transition graph, either all variables that can be changed are changed simultaneously or one variable is changed one at a time. The first case is referred to as a synchronous updating strategy, whereas the second is called the asynchronous updating strategy. In our case, we choose to simulate our models using the asynchronous strategy.

Solutions of a logical model: We define the attractors of the model as the long-term asymptotic behaviors of the system. Two types of attractors are identified: stable states, when the system has reached a model state whose successor in the transition graph is itself; and cyclic attractors, when trajectories in the transition graph lead to a group of model states that are cycling.

If the asynchronous updating strategy is chosen Figure S2, the toy model presents two types of attractors: a stable state and a limit cycle depending on the value of C. There are two disconnected components of the state transition graph for this example that correspond to the two possible values for the input C. If C is initially OFF (equal to 0), then there exists only one stable state: 000. All the trajectories in the state transition graph lead to one final model state (Figure S2 left). If C is initially ON (equal to 1), then the attractor is a limit cycle. The path in the state transition graph cycles for any initial model state of this connected component (Figure S2 right).

The same two solutions are obtained using synchronous updates (Figure S3, where the dashed edge accounts for two changes in the model states. Note that for the asynchronous and synchronous update modes, the stable states solutions of the model are the same. In some cases, some differences may appear between the two update modes concerning the limit cycles.

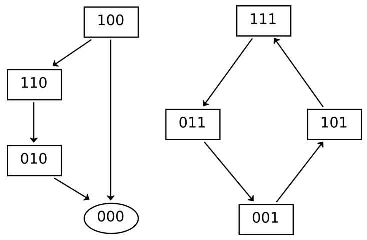

Figure S2: Asynchronous state transition graph corresponding to the toy model presented in Figure S1.

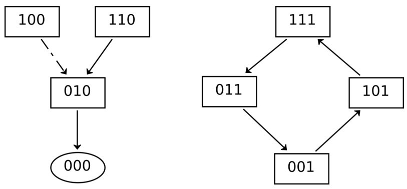

Figure S3: Synchronous state transition graph corresponding to the toy model presented in Figure S1. The dashed edge represents two changes in the model state (A: 1 to 0 and B: 0 to 1).

Markovian simulations: In present work we have used MaBoSS software, that uses Gillespie algorithm. This algorithm is particularly useful when the state transition graph is too big, as it allows to stochastically sample trajectories from a given initial condition to all possible asymptotic solutions and associate a probability to each model state and final stable states.

Using our toy example, for any initial condition (C is either 0 or 1), we find that there is one stable state solution (000 termed $< nil >$ in Figure S4) with a 50% probability. Meaning that half of the trajectories for this initial condition ended in the 000 stable state solution. We also confirm the existence of a limit cycle (damped oscillations) for all model states with an active C (ABC equivalent to 111, AC equivalent to 101, BC equivalent to 011, and C equivalent to 001), which correspond to the nodes that belong to the limit cycle solution in Figures S2 and S3.

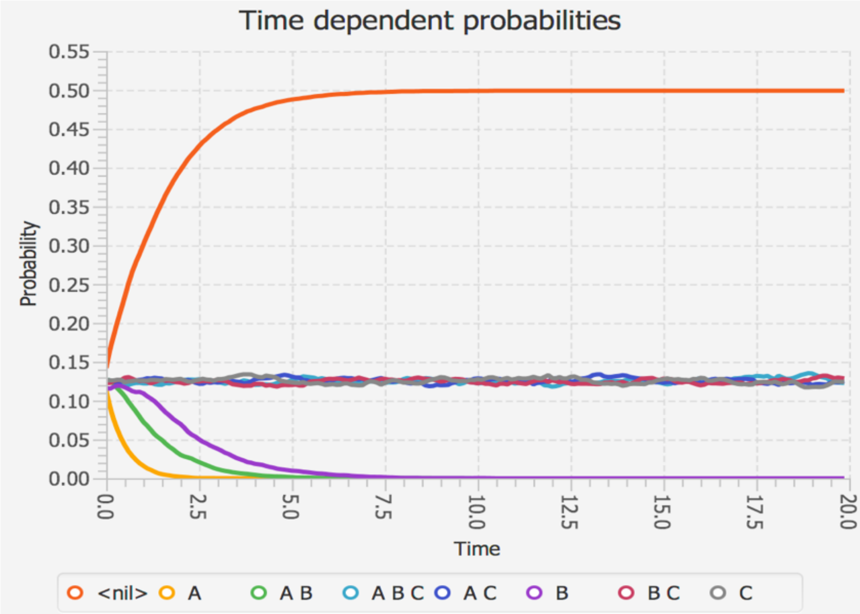

Figure S4: Time dependent probabilities of the MaBoSS simulation of the toy model.

1.2 MaBoSS principles

For the sake of completeness, this section is partially redundant with the summarized description of MaBoSS in the main article. Several logical modeling frameworks and simulation methods have been used to solve cellular biology problems, for thorough reviews, please refer to Abou-Jaoudé et al. (2016) and Wang et al. (2012). In present study, all simulations have been performed with MaBoSS (that stands for **Ma**rkovian **Bo**olean **S**tochastic **S**imulator) (Stoll et al., 2012, 2017). This framework is based on an asynchronous update scheme combined with a continuous time feature obtained with Gillespie algorithm (Gillespie, 1976), allowing simulations to be continuous in time despite the discrete nature of logical modeling.

Gillespie algorithm provides a stochastic way to choose a specific transition among several possible ones and to infer a corresponding time for this transition (Figure S5). Thus, MaBoSS computation results in one stochastic trajectory as a function of time when objective transition rates, seen as qualitative activation or inactivation rates, are specified for each node. These transition rates can be set either all to the same value by default in the absence of any indication or in various levels reflecting different orders of magnitude of biological processes' time: post-translational modifications are quicker than transcriptions for instance. They can also be used to vary speeds depending on inputs or even to adapt multi-valued logical mechanisms in a binary framework (Stoll et al., 2012). These transition rates are translated as transition probabilities in order to determine the actual transition (Figure S5C and D). In present work, all transition states were assigned to 1. All in all, this modeling framework is at the intersection of logical modeling and continuous dynamic modeling.

In present work, nodes or phenotypes probabilities at the asymptotic state are discussed rather than the transient dynamics. Indeed, asymptotic states are more closely related to logical model attractors than transient dynamics and are therefore less dependent on updating stochasticity and more biologically meaningful (Huang et al., 2009).

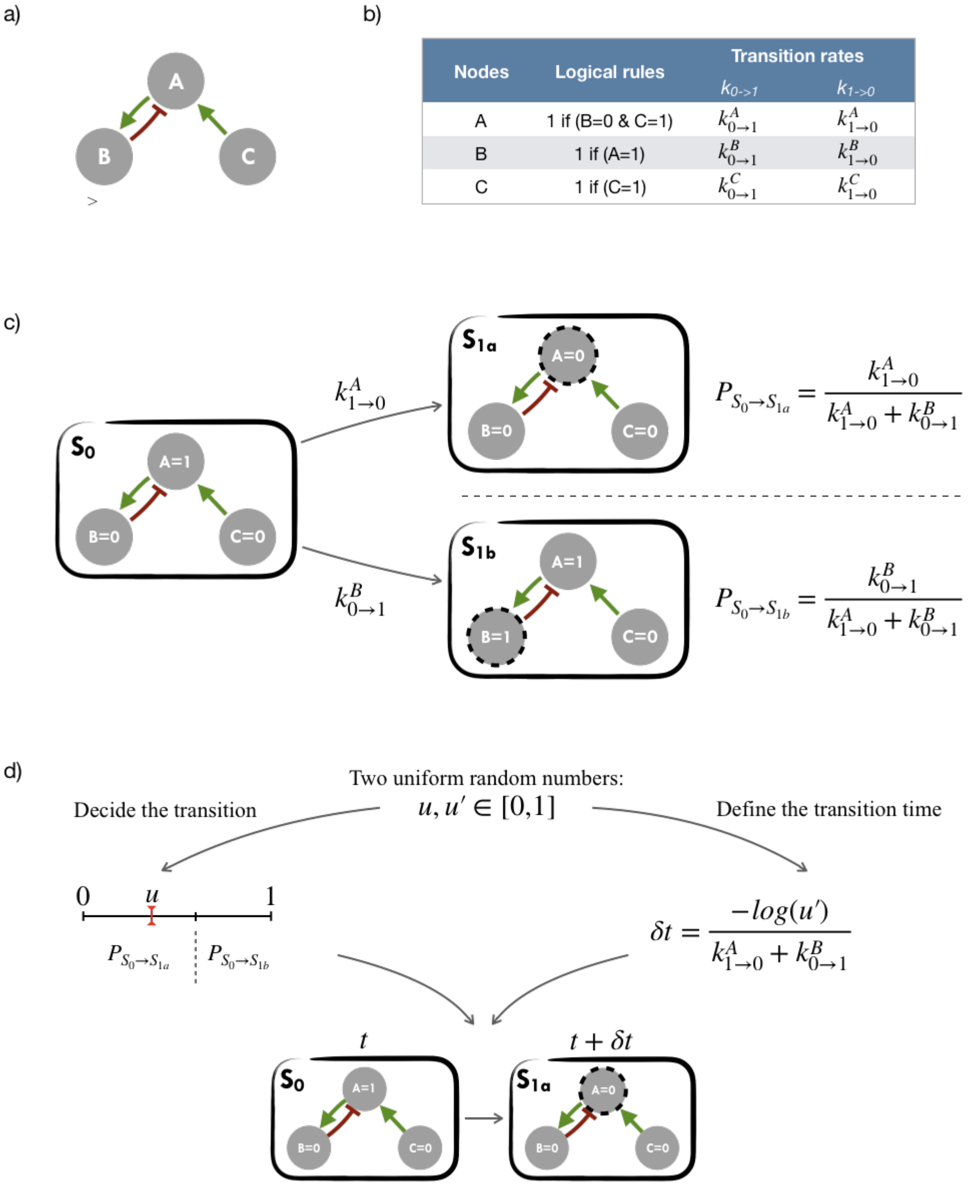

Figure S5: Main principles of MaBoSS simulation framework and Gillespie algorithm, partly reproduced from Figure 2 in main text. (A) Toy model with A regulating B and C, the same that was shown in Figure S1. (B) Table summarizing nodes, logical rules and transition rates of the toy model. In the first column, list of nodes of the toy mode. In the second column, logical rules of the logical model are described: as an input A remains in its initial state; presence of A triggers B activation and C inhibition. In the third column, MaBoSS activation and inactivation transition rates are defined for each transition. (C and D) Example of the use of the transition rates when deciding transitions. (C) In an asynchronous update scheme, starting from S_0 state in t_0 there are two possible resulting states S_{1a} and S_{1b} with their corresponding probabilities. (D) u and u' are respectively used to randomly choose the effective transition (in proportion to the respective probabilities) and the corresponding time of transition, which depends on random u' and the sum of the transition rates.

Since MaBoSS computes stochastic trajectories, it is highly relevant to compute several trajectories in order to get an insight of the average behavior by generating a population of stochastic trajectories over the asynchronous state transition graph. The aggregation of stochastic trajectories can also be interpreted as a description of an heterogeneous population. In present work, all simulations have consisted on the average

of 1000 computed trajectories, but we have nevertheless studied the effect of the number of stochastic trajectories on the studied phenotypes in section 1.6.1.

Since several trajectories are simulated, initial values of each node can be defined with a continuous value between 0 and 1 representing the probability for the node to be defined to 1 for each new trajectory. For instance, a node with a 0.6 initial condition will be set to 1 in 60% of simulated trajectories and to 0 in 40% of the cases.

1.3 Fumiã and Martins model description

For this article, a published Boolean model from Fumiã and Martins (2013) has been used to illustrate our PROFILE methodology. This regulatory network summarizes several key players and pathways involved in cancer mechanisms such as RTKs, PI3K/AKT, WNT/β-catenin, TGF-β/Smads, Rb, HIF-1, p53 and ATM/ATR. An input node *Acidosis* has been added, along with an output node *Proliferation* used as a read-out for the activity of any of the cyclins (*CyclinA*, *CyclinB*, *CyclinD* and *CyclinE*). This slightly extended model contains 98 nodes and 254 edges and its inputs are *Acidosis*, *Nutrients*, Growth Factors (*GFs*), *Hypoxia*, *TNFalpha*, *ROS*, *PTEN*, *p14ARF*, *GLI*, *FOXO*, *APC* and *MAX*; it's outputs are *Proliferation*, *Apoptosis*, *DNA repair*, *DNA damage*, *VEGF*, *Lactic acid*, *GSH*, *GLUT1* and *COX412*.

This model, formatted in MaBoSS format files, is available in our GitHub repository: (https://github.com/sysbio-curie/PROFILE/tree/master/Models/Fumia2013). All logical rules can be found in the model description file (.bnd).

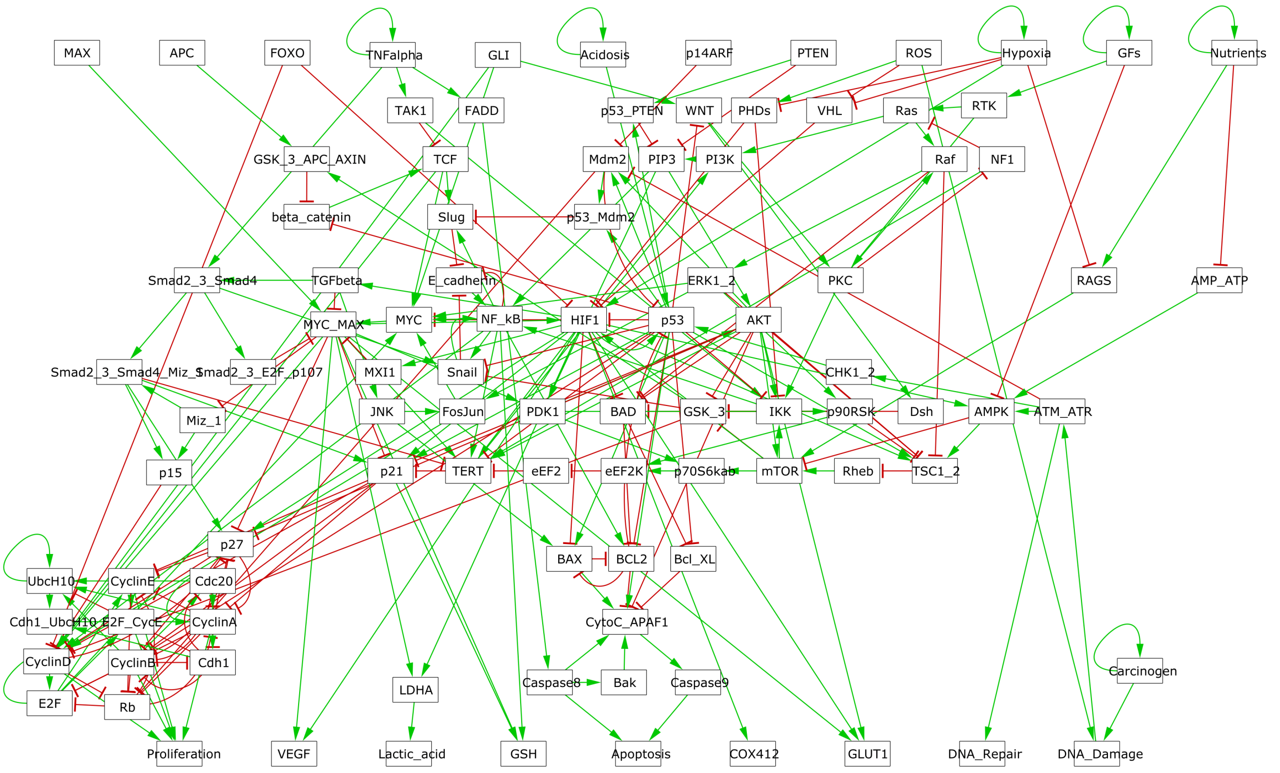

Figure S6: Fumiã and Martins model network. Green edges correspond to positive influences of one node onto its target, and red edges to inhibitory interactions. The network is visualized using GINsim software

In present work, we have focused our analyses on outputs *Proliferation* and *Apoptosis*. For a complete study of the resulting fixed points and limit cycles of this model, we refer the reader to the initial publication Fumiã and Martins (2013).

1.4 Identifying bimodal patterns in genes' distributions across cohort in METABRIC data

Dip test (Hartigan and Hartigan, 1985), bimodality index (Wang et al., 2009) and kurtosis (Teschendorff et al., 2006) criteria might seem as similar tools to select genes whose values can be clustered in two distinct groups of comparable size, but in PROFILE we chose to combine them in order to correct their respective limits and increase the robustness of our method. This section dwells on the reasons to combine them and the selected criteria, continuing the discussion on section 2.3.2.1 and Figure 3 of main text.

In short, for a gene to be be considered as bimodal, it had to fulfill conditions to all three tests. As we have described in the main document, different thresholds were needed to be fulfilled for each test. We have used thresholds proposed by the authors of each of the statistical methods, or else we chose the ones widely used in the community: a dip test result under 0.05, a bimodality index under 1.5 and a kurtosis under 1.

In order to show the relative differences and complementarities between these criteria and showcase different genes' distributions that fulfilled one, two or all of these conditions, Figure S7 shows some genes' distributions and their tests' results. RPL9 is the only gene that fulfills our bimodal test and thus is considered bimodal. All other seven genes are considered as non-bimodal and are further tested to be either considered as unimodal genes (such as AKT2) or as zero-inflated genes (such as KDM5D or INSL6).

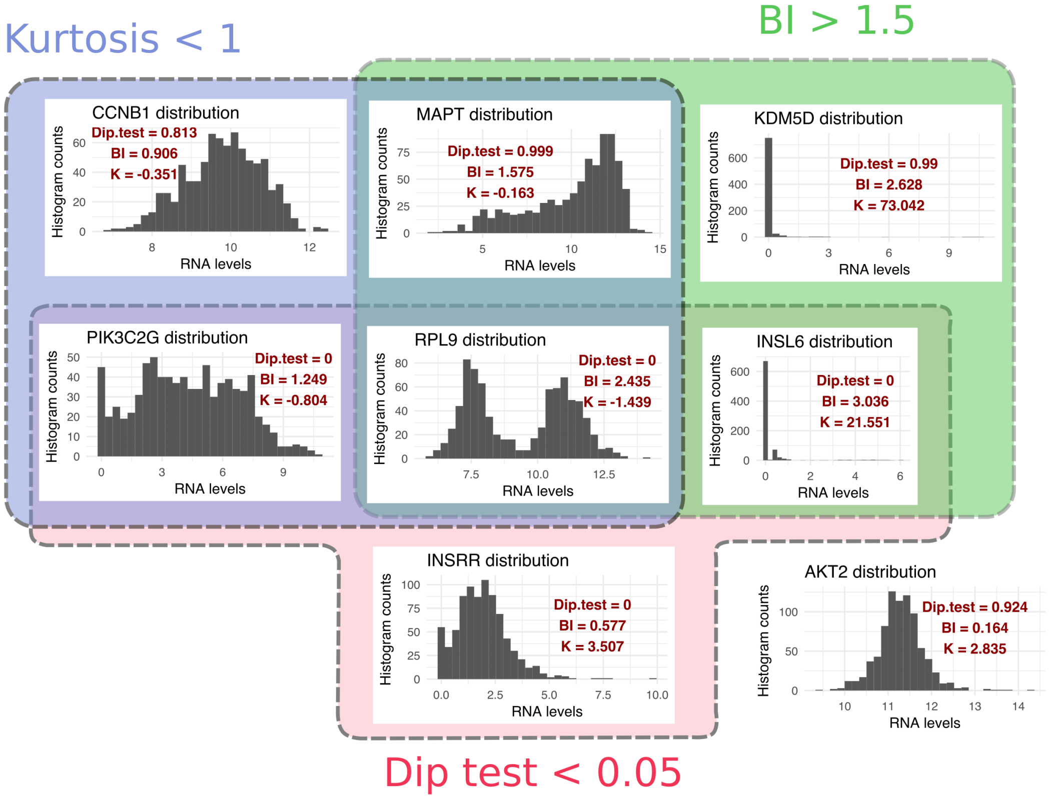

Figure S7: RNA levels distributions for 9 genes and their Hartigan's dip test of unimodality, Bimodality Index (BI) and kurtosis results. Histograms in colored areas have a positive result to the corresponding test. Therefore, RPL9 fulfills all three tests and is considered as bimodal, all others are considered not bimodal.

1.5 Comprehensive description of binarization and normalization methods

The preliminary classification of gene distributions as bimodal, unimodal or zero-inflated enables both their normalization and binarization with similar methods. This section is an extension of the topics discussed in section 2.3.2.1 and Figure 4 of main text. Binarization and normalization functions are thus defined as follows:

$$\begin{aligned} Bin\colon OriginalValues &\to BinarizedValues \\ X &\mapsto Bin(X) \\ Norm\colon OriginalValues &\to NormalizedValues \\ X &\mapsto Norm(X) \end{aligned}$$

Bimodal genes processing: Gaussian mixture models

In PROFILE, a 2-component Gaussian mixture model is fitted using `mclust` R package resulting in a lower mode M_0 and a upper mode M_1 (Figure S8). Each data point X has a probability to belong to M_0 or M_1 such as

$$Prob(X_{gene_i,sample_j} \in M_{0,gene_i}) + Prob(X_{gene_i,sample_j} \in M_{1,gene_i}) = 1 \tag{S1}$$

For these bimodal genes, binarization and normalization processing is defined as:

$$Bin(X_{gene_i,sample_j}) = \begin{cases} 1 & \text{if } Prob(X_{gene_i,sample_j} \in M_{1,gene_i}) \geq 0.95 \\ 0 & \text{if } Prob(X_{gene_i,sample_j} \in M_{0,gene_i}) \geq 0.95 \\ NA & \text{otherwise} \end{cases} \tag{S2}$$

$$Norm(X_{gene_i,sample_j}) = Prob(X_{gene_i,sample_j} \in M_{1,gene_i}) \tag{S3}$$

Non-bimodal gene binarization: outliers' assignment

Since these distributions have no clear bimodal pattern, we choose to binarize only some extreme samples for these genes and leave the others unassigned. In PROFILE, for each gene, we binarize only outliers of the distribution across the whole cohort using the inter-quartile range, a widely acknowledged robust estimator of dispersion, that is less sensitive to outliers (Tukey, 1977). For each gene, we define the inter-quartile range as:

$$IQR_{gene_i} = q_{75,gene_i} - q_{25,gene_i} \tag{S4}$$

where $q_{25,gene_i}$ and $q_{75,gene_i}$ are respectively the first and third quartiles of $gene_i$ expression data distribution. Similar to outlier-sum statistic (Tibshirani and Hastie, 2007), binary assignments are derived as follows:

$$Bin(X_{gene_i,sample_j}) = \begin{cases} 1 & \text{if } X_{gene_i,sample_j} \geq q_{75,gene_i} + IQR_{gene_i} \\ 0 & \text{if } X_{gene_i,sample_j} \leq q_{25,gene_i} - IQR_{gene_i} \\ NA & \text{otherwise} \end{cases} \tag{S5}$$

This method is applied to both unimodal and zero-inflated genes (Figure S8).

Unimodal Gene Sigmoid Normalization

For unimodal distributions, in PROFILE we transform data using a sigmoid function to maintain the most common pattern which is unimodal and nearly-symmetric (Figure S8). First of all, expression data are centered around the median, which is more robust against outliers than using the mean:

$$X'_{gene_i,sample_j} = X_{gene_i,sample_j} - median_{gene_i}(X) \tag{S6}$$

Then data are normalized through the sigmoid function:

$$Norm(X'_{gene_i,sample_j}) = \frac{1}{1 + e^{-\lambda.X'_{gene_i,sample_j}}} \tag{S7}$$

Since the slope of the function depends on λ, we adapt λ to the dispersion of the initial data in order to maintain a significant dispersion in $[0, 1]$ interval: more dispersed unimodal distributions are mapped with a gentle slope, sharply peaked distributions with a steep one. We map the median absolute deviation (MAD) on both sides of the median respectively to 0.25 and 0.75. First, the MAD is defined as:

$$MAD_{gene_i}(X) = median(\mid x_i - median_{gene_i}(X_{gene_i,sample_j}) \mid) \tag{S8}$$

Therefore, to fulfill the proposed mapping, we solve:

$$\frac{1}{1 + e^{\pm\lambda.MAD}} = \frac{1}{2} \mp \frac{1}{4}, \tag{S9}$$

and derive:

$$\lambda = \frac{log_e(3)}{MAD}. \tag{S10}$$

Thus, we obtain data normalized in $[0, 1]$ for unimodal genes, as in Figure S8

Zero-inflated genes sigmoid normalization

Zero-inflated genes are characterized by a distribution density peak close to 0 (Figure S8). In PROFILE, we compute this using the `density` function of `stats` R package. For this case, we linearly transform the initial distribution in order to maintain the asymmetric original pattern:

$$Norm(X_{gene_i,sample_j}) = \frac{X_{gene_i,sample_j} - min_{gene_i}(X)}{max_{gene_i}(X) - min_{gene_i}(X)} \tag{S11}$$

The transformation is applied to data between 1st and 99th quantiles to be more robust to outliers. Values below q_1 or above q_{99} are respectively assigned to 0 and 1.

1.6 Simulation parameters and personalization methods

1.6.1 Influence of the number of stochastic trajectories to MaBoSS results

State transition graph, or STG, scales exponentially to the size of the model's network. For instance, in a logical model with 100 nodes, the number of model states can be of 2^{100}. In PROFILE, we use

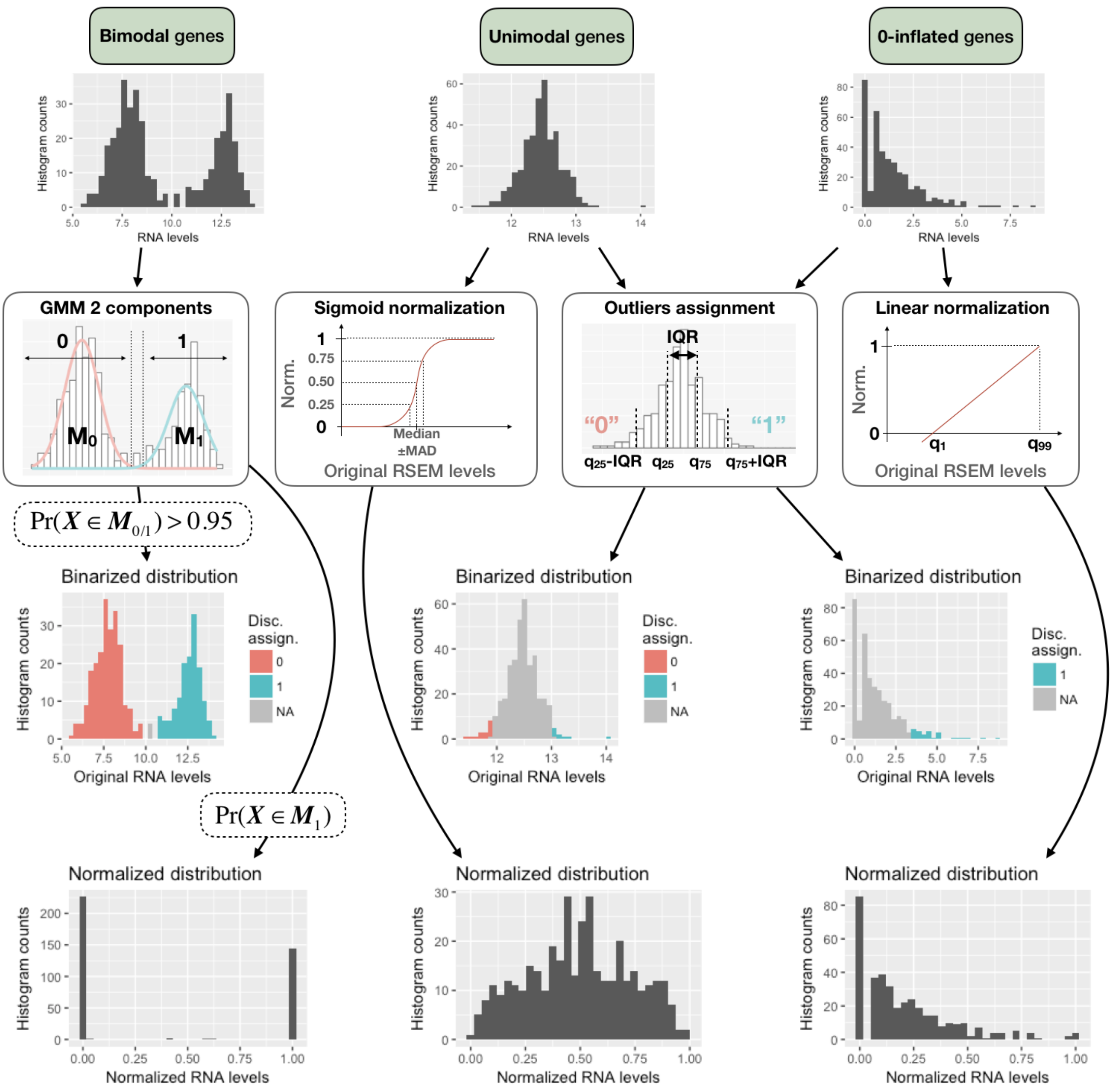

Figure S8: Binarization and normalization methods for expression data of genes of all three categories (bimodal, unimodal and zero-inflated). Top row panels show examples of original patterns from genes of the three categories. Second row panels illustrate the processing methods used for both binarization and normalization (GMM 2-component, sigmoid normalization, outliers assignment and linear normalization). Third row panels correspond to the result of using the binarization methods on these genes and fourth row panels of using normalization methods.

MaBoSS that uses Gillespie algorithm to explore the STG with stochastic trajectories. The bigger the graph, the more potential stable states it will have and the fewer trajectories will reach these stable states. Thus, it is important to verify that the number of simulated stochastic trajectories to explore the STG is sufficient to properly reach these stable states, meaning that the probability to reach them that does not have a high variability. Using METABRIC cohort we investigated the results of RNA-based transition rates personalization with different number of stochastic trajectories (1000, 2000 and 5000 trajectories) and with

10 replicates each (Figure S9). The standard deviation of *Proliferation* and *Apoptosis* nodes' probabilities was analyzed as they were the focus of the paper.

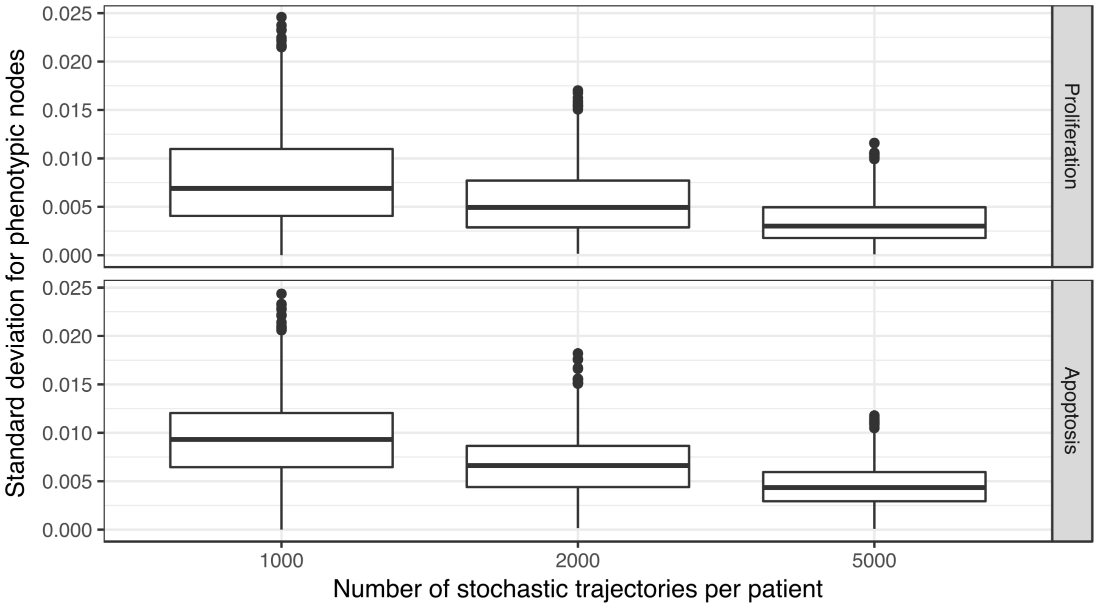

Figure S9: Robustness analysis of *Proliferation* and *Apoptosis* phenotypic probabilities with varying number of stochastic trajectories in MaBoSS simulation. 10 replicates were performed for each METABRIC patient with RNA data (1904 patients) and their patient-specific standard deviations calculated.

We built personalized models for each METABRIC patient with RNA information (n=1904) using RNA as *Soft Node Variants* as explained in section 3.2 and Figure 6, case 4, of the main text. 10 replicates were computed for each number of stochastic trajectories and their patient-specific standard deviation was calculated. As it can be seen in Figure S9, the standard deviations decreases as the number of trajectories increases, but 1000 trajectories are sufficient to have a median deviation lower than 0.01. Therefore we have selected to use 1000 trajectories in our simulations. Nevertheless, we encourage MaBoSS users to perform such an analysis and check that the number of trajectories used allows for low standard deviations in their analyses.

1.6.2 Influence of the amplification factor when personalizing transition rates

As discussed in section 2.4.3 of the main text, when personalizing the logical models' transition rates in PROFILE framework, a user-defined amplification factor (AF) parameter is used to generate a higher relative difference in the transition rates. In order to select a parameter value that would yield the most meaningful results, we performed analyses to assess its influence on the results (Figure S10).

Small AFs have little influence on simulations and nodes' probabilities have a very narrow distribution around a peak corresponding to non-personalized models' probability. Conversely, high AFs deeply impact the models and result in spread distributions (Figure S10A). To correlate the different simulated probabilities with a classification score, we compared them to the RNA-based "G2M Checkpoint" and "Apoptosis" Hallmarks' signatures from MSigDB (Liberzon et al., 2015). It can be seen in Figure S10B that improved correlations are seen with higher AFs with a phenotype-specific plateau effect. We had to compensate our desire of using a high enough AF as to have a good correlation with the signatures with our inclination of not having completely flat probabilities' distributions. Therefore, we decided to set AF as

100, as it is at the beginning of the plateau, following a strategy similar to the elbow criterion, and it does not produce flat phenotypes' probabilities' distributions.

a)

b)

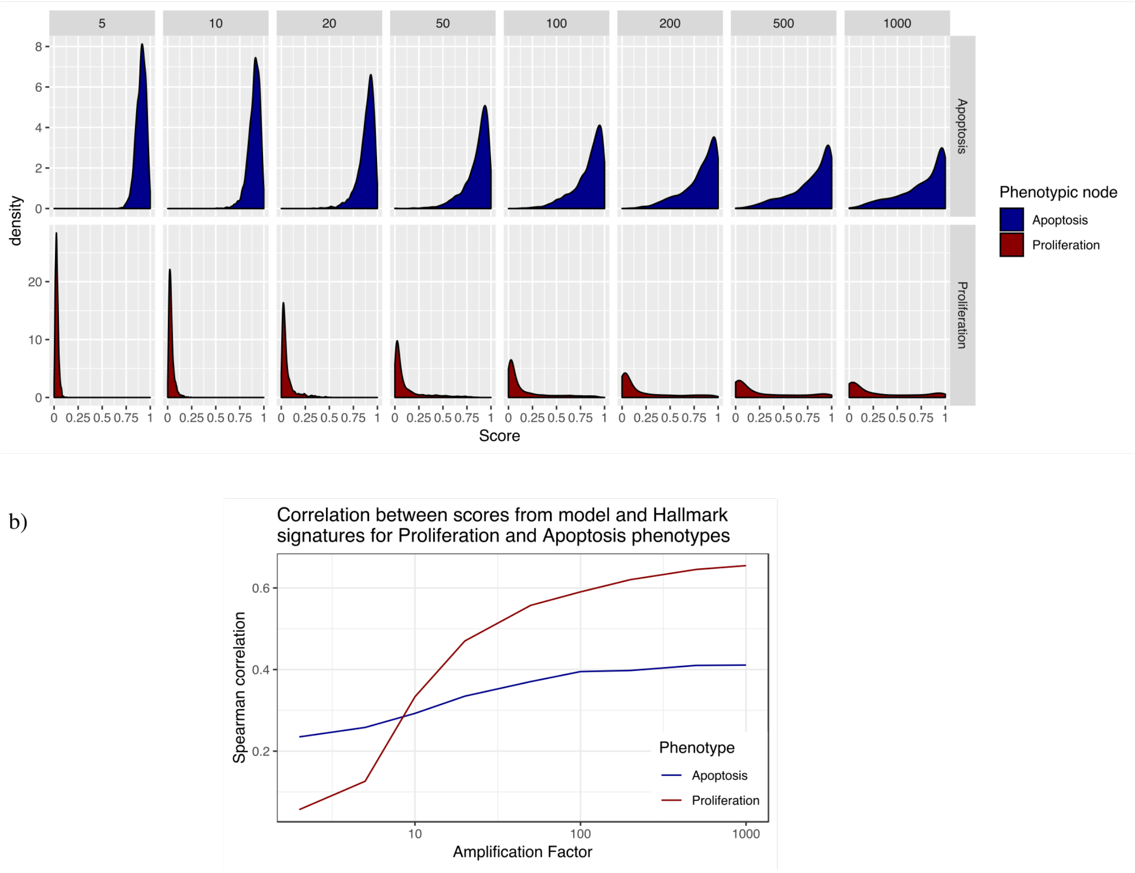

Figure S10: Influence of amplification factor on simulations results. (A) Distribution of *Proliferation* and *Apoptosis* model probabilities in the METABRIC cohort with different amplification factors. (B) Correlation between *Proliferation* and *Apoptosis* model probabilities to RNA-based "G2M Checkpoint" and "Apoptosis" Hallmarks' signatures scores for different amplification factors.

1.7 Distribution of data types samples in METABRIC and TCGA datasets

Patient's data from METABRIC (Curtis et al., 2012; Pereira et al., 2016) and TCGA (Cancer Genome Atlas Network, 2012; Ciriello et al., 2015) were used in PROFILE to perform different analyses, not always the same for all of them. For instance, in METABRIC more patients had their exome analyzed (2509) than their transcriptomics (1904) (Figure S11A). In our different personalization protocols we used as much information we could gather in a method-specific manner: if the personalization used mutation profiles and CNA data in METABRIC, the 2173 patients with data of these types were considered.

Additionally, METABRIC's number of records are three times bigger than TCGA's (Figure S11B). As this divergence could impact the comparison of results among those two datasets, in present work we have analyzed dataset-specific personalizations that use different data from the same dataset, without cross-analyzing METABRIC and TCGA datasets.

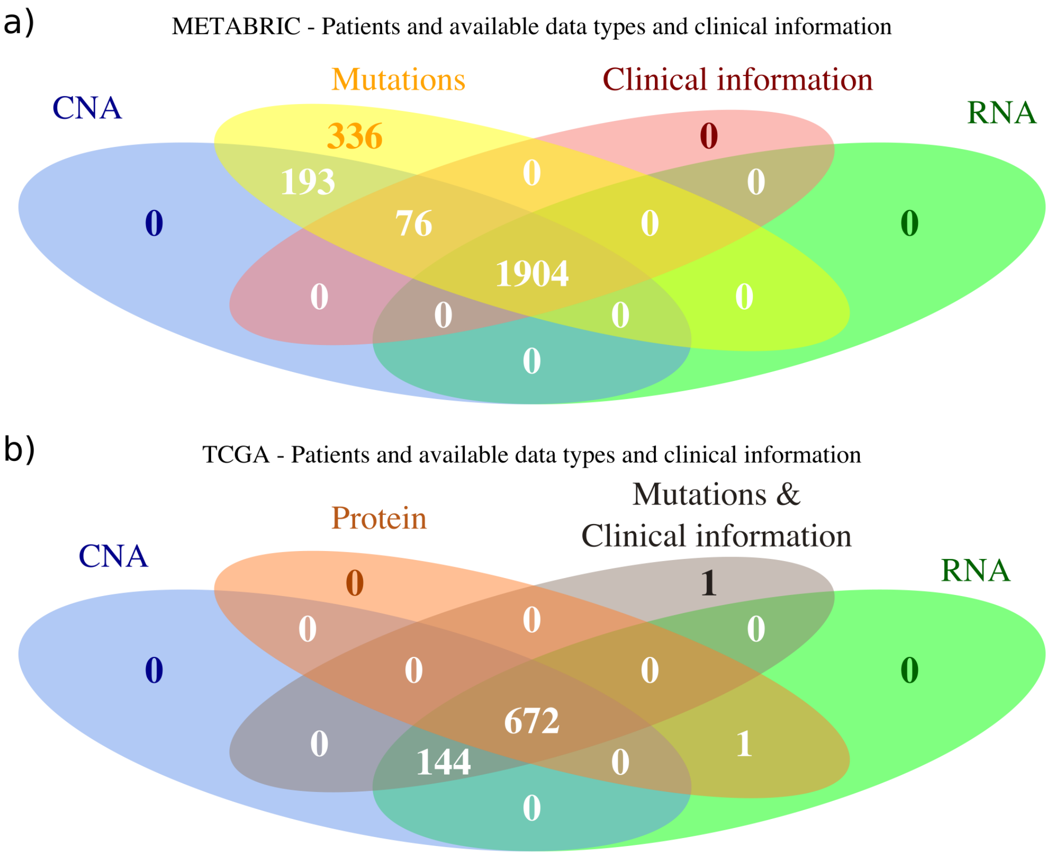

Figure S11: Distribution of data types (omics and clinical) available for each subset of patients in METABRIC (A) and TCGA (B) data bases.

2 SUPPLEMENTARY RESULTS

2.1 Using the personalization framework with other models and data

In present work, we have shown results of our PROFILE framework using Fumiã and Martins (2013) generic cancer model on METABRIC data. Nevertheless, Fumiã and Martins (2013) logical model does not take into account key genes in breast cancer progression such as hormone receptors and their associated signaling networks. Thus, we decided to use PROFILE on an alternative breast-cancer-specific model (Zañudo et al., 2017) together with the same METABRIC dataset. In Figure S12 it can be seen the different personalized patient- and breast-specific models results obtained that had similar results to Fumiã's patient-specific model ones (section 3.2 and Figure 6 of the main text).

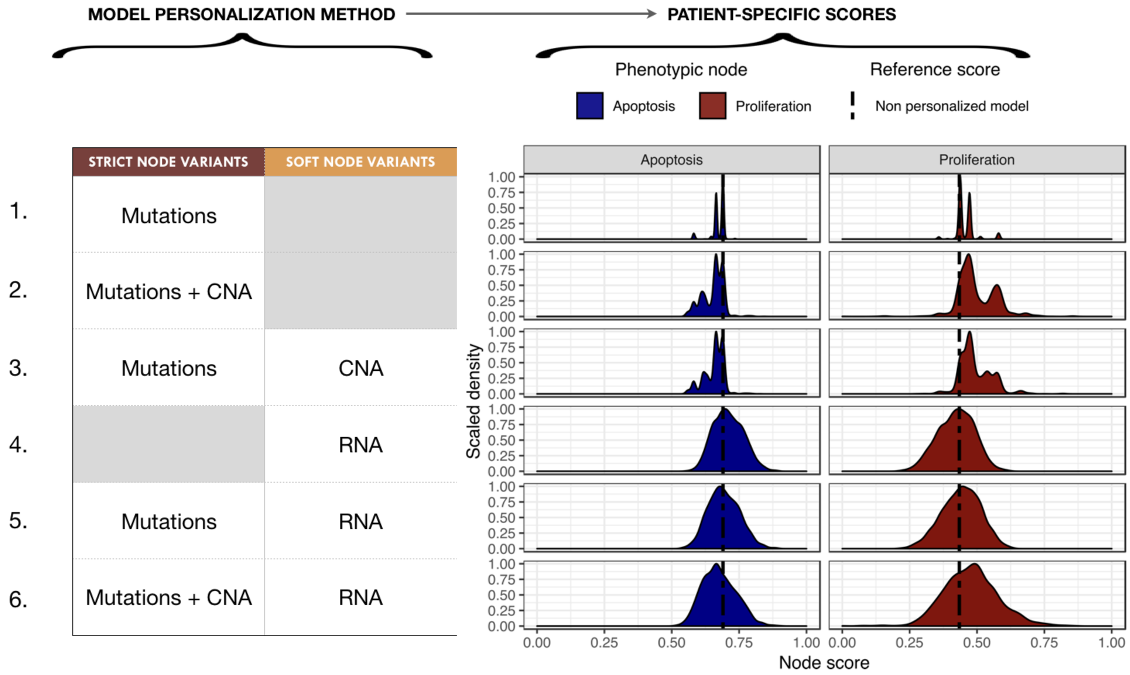

Figure S12: Impact of different model personalization methods on the distribution of phenotypic nodes *Proliferation* and *Apoptosis*, using a breast-specific cancer model and METABRIC data. On the left, description of data types used as *Strict* or *Soft Node Variants* to personalize the model resulting in different phenotypic probabilities' distributions across the cohort, as shown on the right. Dashed lines correspond to the probabilities of the phenotypes obtained from the original model without any personalization: 0.019 for *Proliferation* and 0.906 for *Apoptosis*.

Similar generic patterns were observed as in Fumiã's model cases: integrating data as *Strict Node Variants* resulted in narrow-peaked distributions (such as case 1 of Figure S12 and case 1 of Figure 6 of the main text) and integrating data as *Soft Node Variants* resulted in smoother distributions (such as case 4 of Figure S12 and case 4 of Figure 6 of the main text). Note that when using *Strict Node Variant* method nodes are set to a given value for the whole simulation, while when using *Soft Node Variant* initial states and transition rates modifications are combined (more on this in section 2.4.4 of the main text).

Nevertheless, this breast-specific model results in narrower distributions around the non-personalized values meaning that this model does not generate more diverse clusters of patients compared to simulations with Fumiã and Martins (2013) generic model. This can be explained by the smaller size of the model, which does not necessarily allow all the information to be captured despite the presence of a few breast-specific pathways. For instance, due to the particular objective of the study of Zañudo et al. (2017), *i.e.*,

investigating drug resistance in breast cancer, well-known cancer players like p53 have not been included in the model.

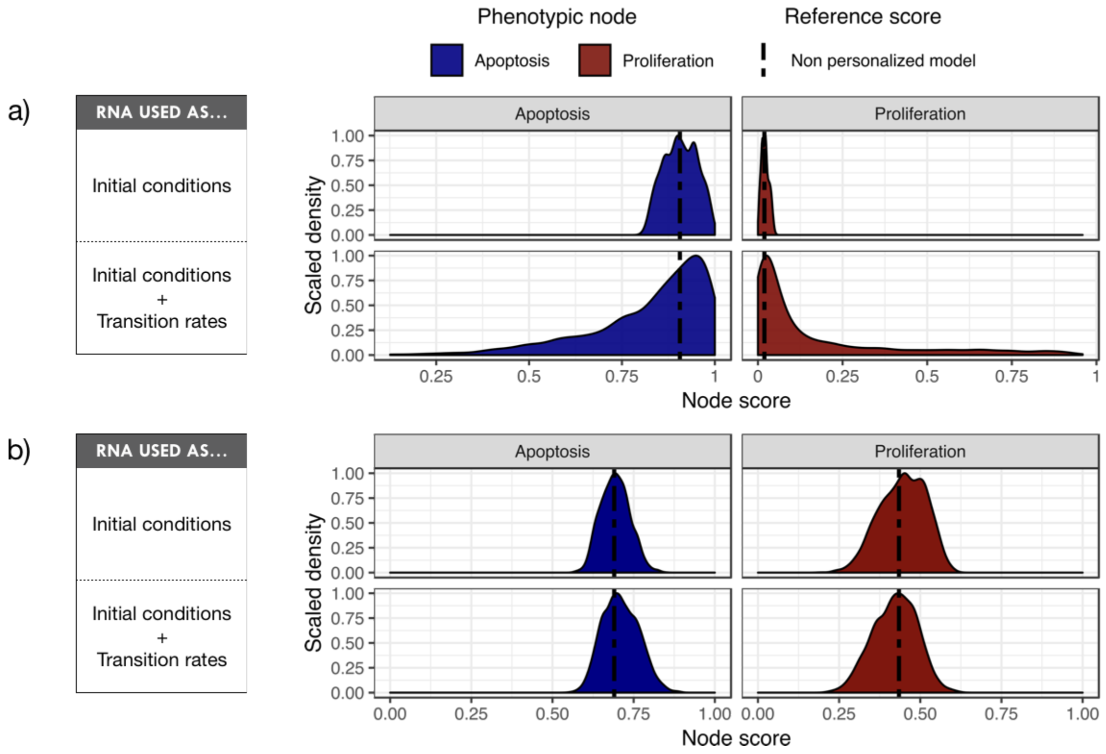

Figure S13: Impact of RNA used as initial conditions alone or as *Soft Node Variants* to personalize Fumiã's generic (A) and Zañudo's breast-specific (B) models. Dashed lines correspond to the probabilities of the phenotypes obtained from the original model without any personalization: 0.019 for *Proliferation* and 0.906 for *Apoptosis*.

For completeness, we explored the comparison of these models using initial conditions and found that the personalization method using those resulted in no significant discrimination between patients with the Fumiã's generic model compared to the Zañudo's breast-specific one (Figure S13). This can be explained by the difference in input nodes (nodes without regulation that maintain their initial state throughout the entire simulation) between these two models: Fumiã's generic one has only environmental variables as input nodes (like *Hypoxia* or *Nutrients*) that have no link to any gene available in the datasets; on the opposite, Zañudo's breast-specific model has known genes or proteins as input nodes (*HER2*, *ER*, *PTEN*). Thus, as initial conditions were determined by the RNA levels, the intersection of genes that had RNA data and were related to input nodes was higher in Zañudo's than in Fumiã's model.

Additionally, we have also expanded our framework to a different dataset: TCGA dataset, which is smaller than METABRIC, but includes protein data (Figure S11). It is important to note that the use of RPPA data requires additional attention. Some antibodies target phospho-sites whose phosphorylation can be either an activation or an inactivation. It is therefore necessary to study the impact of phosphorylation on the regulations described in the model. This study can be done through a review of the literature or existing databases. The files in our GitHub provide an example on how to use these RPPA data with the interpretation of phosphorylations.

Using Fumiã's model and TCGA dataset, we see that phenotypes' distributions are different when using different personalizations methods (Figure S14), but are nonetheless comparable to the results using

	STRICT NODE VARIANTS	SOFT NODE VARIANTS
1.	Mutations	
2.	Mutations	CNA
3.		RNA
4.		RNA (Healthy samples)
5.		RNA + RPPA
6.	Mutations	RNA
7.	Mutations	RNA + RPPA
8.	Mutations + CNA	RNA
9.	Mutations + CNA	RNA + RPPA

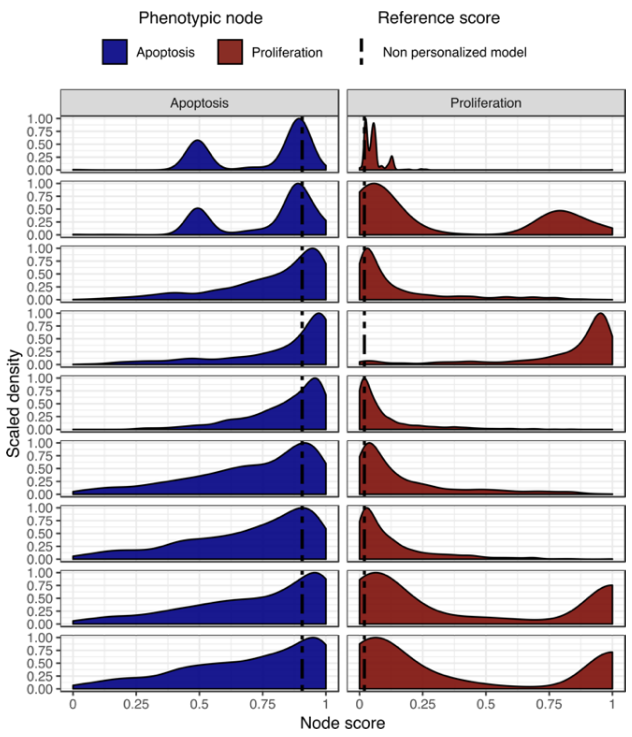

Figure S14: Distribution of *Proliferation* (left) and *Apoptosis* (right) model probabilities with different personalization methods, using Fumiã's cancer model and TCGA data. Dashed lines are results of the original model without any personalization.

Fumiã's model and METABRIC data (Figure 6 in the main text). When RNA and RPPA are used together, the nodes are linked first to the RPPA data and, in the absence of the former, to the RNA data. This is necessary duet to the small number of nodes with RPPA measures.

Nevertheless, the analyses of these distributions were informative, but did not shed much light to the underlying biology of the problem, which is the reason behind the use of signature's correlation to classify which personalization methods were closer to a validation score.

2.2 Correlation to signatures

In the main text, we have correlated our different personalizations cases of Fumiã's generic model using METABRIC dataset to a set of molecular signatures and Nottingham Prognostic Index (NPI) (section 3.3 and Figure 7 in the main text). Here, we present PROFILE use for the same analyses using Fumiã's generic model and TCGA dataset (Figure S15). The same global trends are observed when compared to Figure 7 and the addition of RPPA data does not cause any performance gains (Figure S15, cases 5, 7 and 9). Note that strategies using RPPA data are implemented on fewer patients than in the other cases (see Figure S11B).

Interestingly, using healthy samples as reference to normalize RNA results in bigger correlations (Figure S15, case 4). This strategy is described with more details in section 2.4.

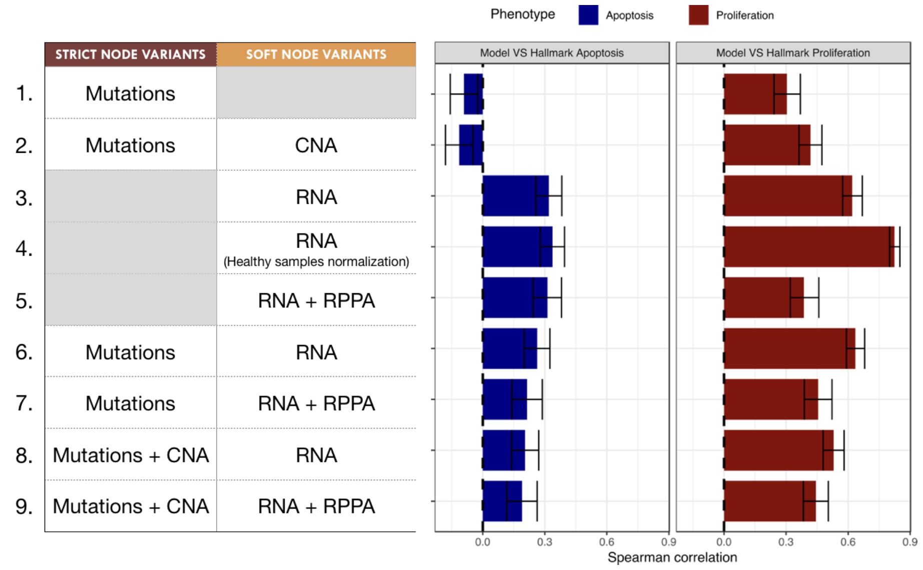

Figure S15: Biological and clinical classification of phenotypic probabilities from personalized models. Model personalization methods used (left) and the corresponding Spearman rank correlation between phenotypic probabilities from personalized model and the corresponding Hallmark signatures scores based on RNA gene sets (right).

2.3 Survival analyses of other personalization cases

To complete the survival analyses presented in the main text, we used PROFILE to perform it for Fumiã's generic model results using METABRIC dataset and case 4 personalization method (RNA expression data used as *Soft Node Variants*).

In this case, *Proliferation* remains very significantly correlated with survival data but *Apoptosis* is not anymore (Figure S16). As we can see, for this analysis, the addition of mutations and/or CNA data is required to recover significant and meaningful correlations.

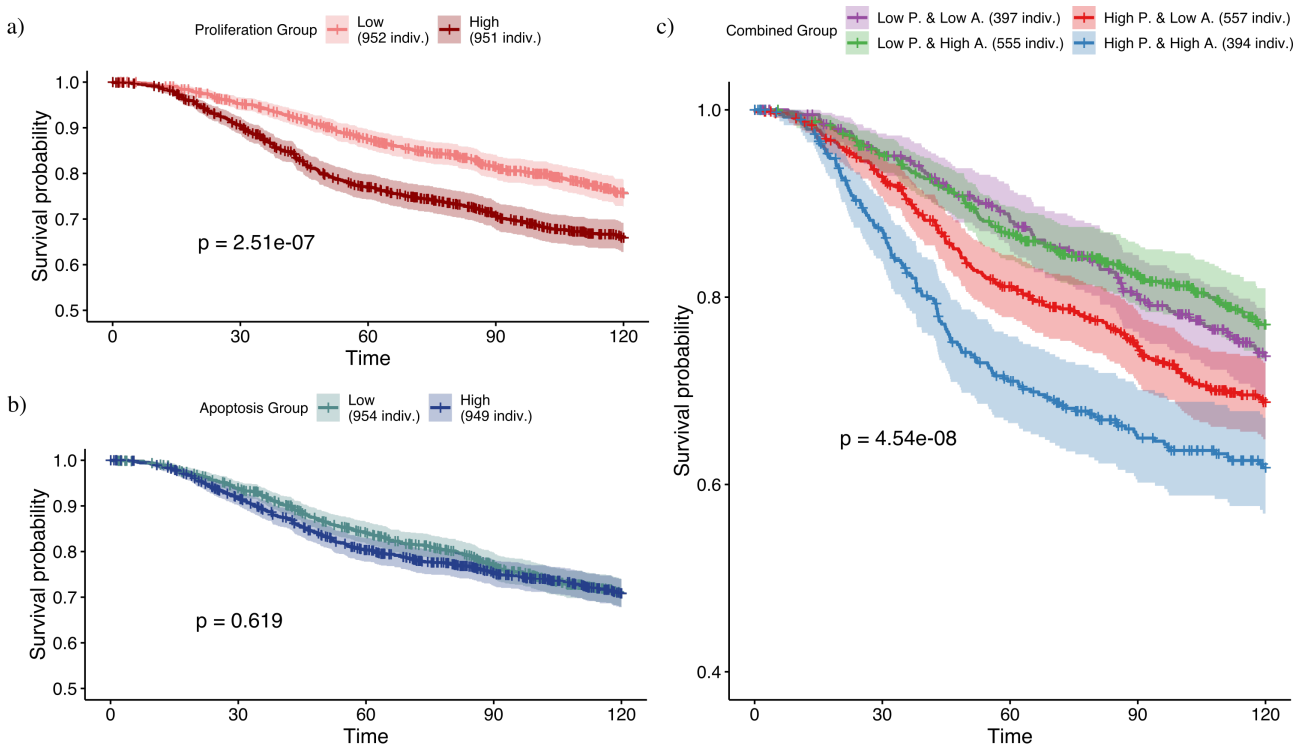

Figure S16: Survival analysis of METABRIC samples from which normalized RNA levels were used as *Soft Node Variants* in the model. All p-values are derived from a log-rank test. (A) Survival curves with high and low *Proliferation* groups. (B) Survival curves with high and low *Apoptosis* groups. (C) Survival curves with combined groups.

2.4 Study on the use of healthy samples in the normalization of RNA data

As it was introduced in section 2.3.2.2 of the main text, the use in PROFILE of corresponding healthy samples of patients to normalize RNA data deserves further investigation. As it can be seen in Figure S15, using Fumiã's model with TCGA dataset with a set of healthy samples for RNA normalization greatly improved the correlation performance of Proliferation to "G2M Checkpoint" signature from Hallmarks of MSigDB.

Nevertheless, the use of this healthy dataset not only improved the correlation performances but also the qualitative trend of the results. Focusing on cases 3 and 4 of Figure S15, we displayed their simulated *Proliferation* probabilities and RNA signature's scores on Figure S17. Using healthy samples instead of cancer samples as reference for RNA normalization resulted in a significant shift of the distribution towards high *Proliferation* model probabilities. Thus, using healthy samples to normalize RNA data, caused the patient-specific models to have much higher *Proliferation* phenotypes, tallying the proliferative behavior of cancerous tissues.

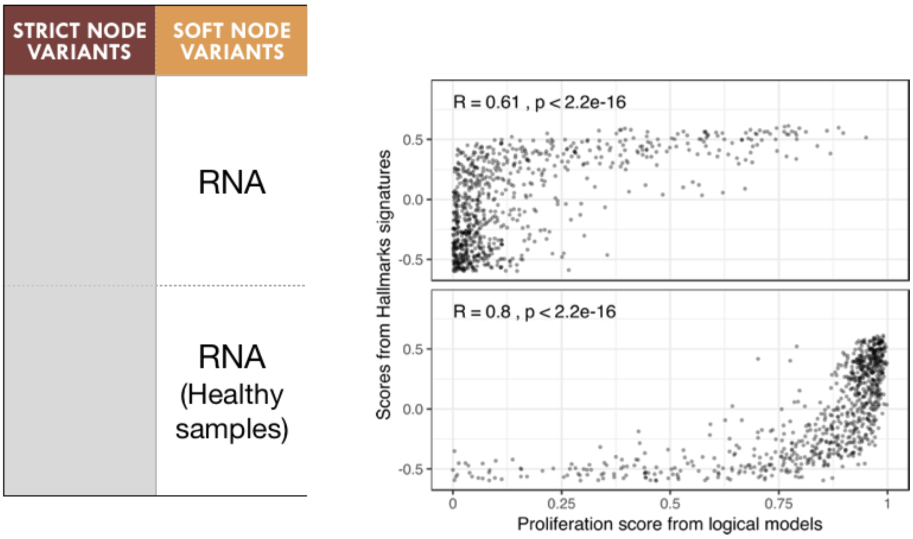

Figure S17: Scatter plot for Spearman rank correlation between Hallmark signature for Proliferation ("G2M Checkpoint", based on RNA data) and *Proliferation* model probabilities using cancer (A) or healthy (B) samples as reference for RNA normalization before personalization with soft node variants.

2.5 Comparing PROFILE and RefBool binarization processes

RefBool (Jung et al., 2017) method for binarization has been investigated to compare it to our PROFILE framework. Using this method, we observe a clear trend towards positive outliers: RefBool appears to be less stringent regarding binarization in 1s, and much more stringent regarding binarization in 0s as shown in Figure S18. RefBool analysis on METABRIC dataset results in 9.5% of binarized values; all of them 1s and not a single 0.

Note that due to the lack of a healthy samples in METABRIC's RNA dataset, we used the RNA dataset as its own reference dataset for normalization: each gene was compared to the distribution of that gene across all samples.

With TCGA dataset, we compared both PROFILE and RefBool methods using healthy samples as a reference for binarization of more than 16 million values (817 samples with 20040 genes). RefBool resulted

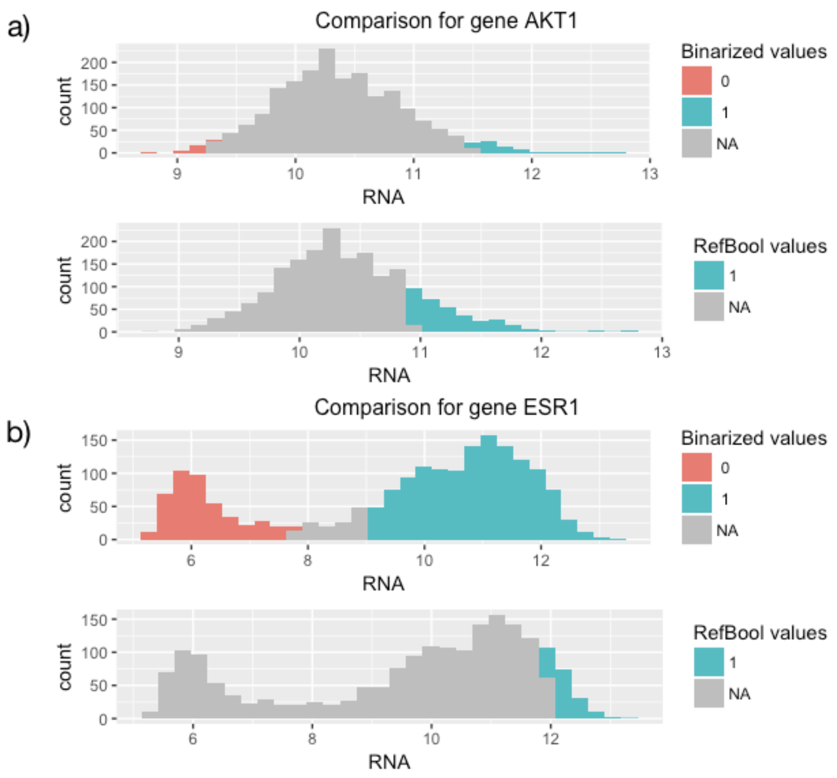

Figure S18: Comparative examples between our PROFILE binarization method for continuous data and RefBool method for a *Unimodal* gene AKT1 (A) and a *Bimodal* gene ESR1 (B) in the METABRIC cohort

in 9.2% of values binarized to 0s and 29.4% binarized to 1s. Our method resulted in 16.2% of values binarized to 0s and 14.4% binarized to 1s. Further evidence to show the clear bias of RefBool towards assigning more 1s than 0s. Examples for some of these genes are shown in Figure S19.

At present, the binarization of RNA data has not been extensively used in our PROFILE framework. In the future, as discussed in the main text, we plan to study the Hamming distance of a binarized profile of patient with each one of the stable states obtained by the non-personalized model.

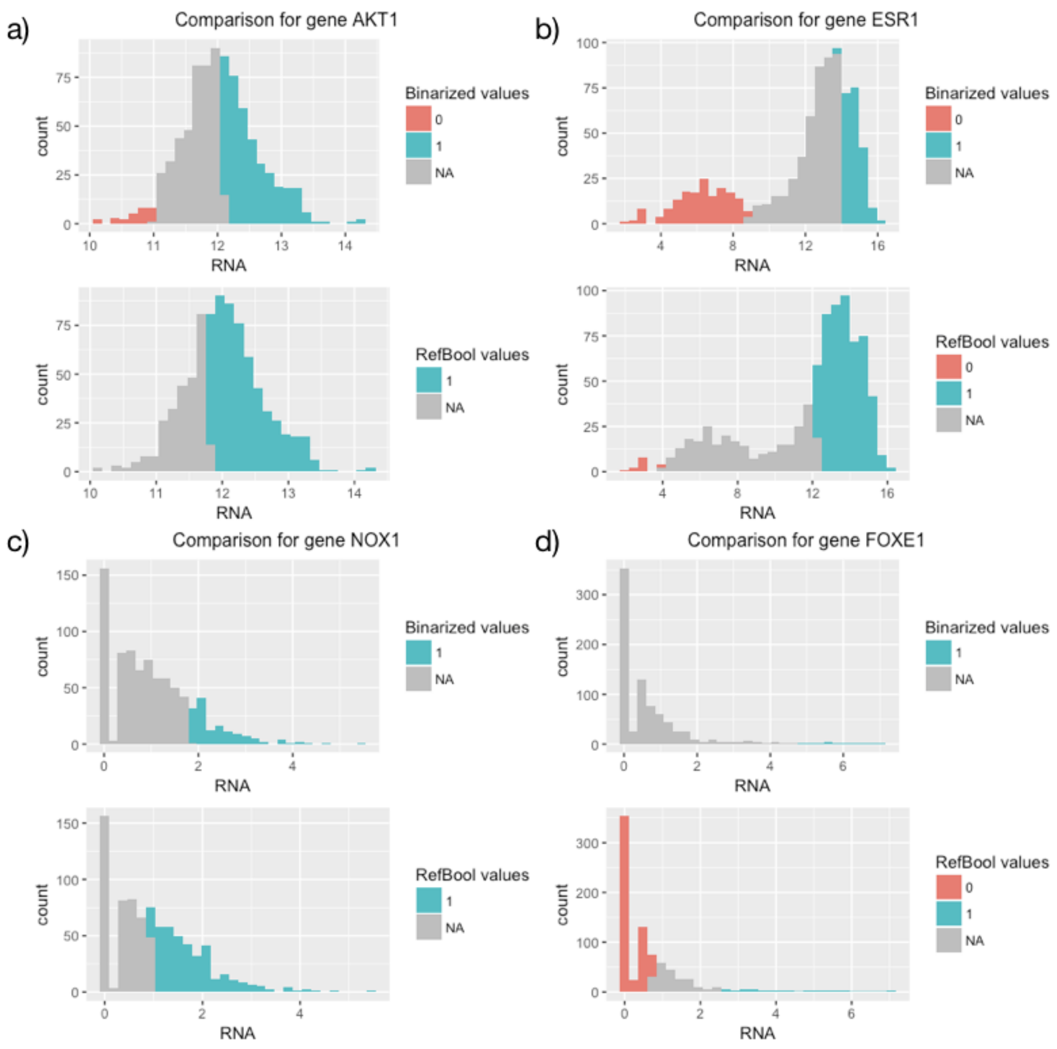

Figure S19: Comparative examples between our PROFILE binarization method for continuous data and RefBool method in TCGA cohort, using healthy samples as reference. A and B correspond to *Unimodal* genes, regarding the genes' distributions in healthy samples, even if in B the distribution for cancer samples is bimodal. C and D correspond to *Zero-inflated* genes, again regarding genes' distribution in healthy samples

REFERENCES

Abou-Jaoudé, W., Traynard, P., Monteiro, P. T., Saez-Rodriguez, J., Helikar, T., Thieffry, D., et al. (2016). Logical Modeling and Dynamical Analysis of Cellular Networks. *Frontiers in Genetics* 7. doi:10.3389/fgene.2016.00094

Calzone, L., Tournier, L., Fourquet, S., Thieffry, D., Zhivotovsky, B., Barillot, E., et al. (2010). Mathematical Modelling of Cell-Fate Decision in Response to Death Receptor Engagement. *PLOS Computational Biology* 6, e1000702. doi:10.1371/journal.pcbi.1000702

Cancer Genome Atlas Network (2012). Comprehensive molecular portraits of human breast tumours. *Nature* 490, 61–70. doi:10.1038/nature11412

Ciriello, G., Gatza, M. L., Beck, A. H., Wilkerson, M. D., Rhie, S. K., Pastore, A., et al. (2015). Comprehensive Molecular Portraits of Invasive Lobular Breast Cancer. *Cell* 163, 506–519. doi:10.1016/j.cell.2015.09.033

Curtis, C., Shah, S. P., Chin, S.-F., Turashvili, G., Rueda, O. M., Dunning, M. J., et al. (2012). The genomic and transcriptomic architecture of 2,000 breast tumours reveals novel subgroups. *Nature* 486, 346–352. doi:10.1038/nature10983

Fumiã, H. F. and Martins, M. L. (2013). Boolean Network Model for Cancer Pathways: Predicting Carcinogenesis and Targeted Therapy Outcomes. *PLoS ONE* 8. doi:10.1371/journal.pone.0069008

Gillespie, D. T. (1976). A general method for numerically simulating the stochastic time evolution of coupled chemical reactions. *Journal of Computational Physics* 22, 403–434. doi:10.1016/0021-9991(76)90041-3

Hartigan, J. A. and Hartigan, P. M. (1985). The Dip Test of Unimodality. *The Annals of Statistics* 13, 70–84. doi:10.1214/aos/1176346577

Huang, S., Ernberg, I., and Kauffman, S. (2009). Cancer attractors: a systems view of tumors from a gene network dynamics and developmental perspective 20, 869–876

Jung, S., Hartmann, A., and Del Sol, A. (2017). RefBool: a reference-based algorithm for discretizing gene expression data. *Bioinformatics (Oxford, England)* 33, 1953–1962. doi:10.1093/bioinformatics/btx111

Kazemzadeh, L., Cvijovic, M., and Petranovic, D. (2012). Boolean model of yeast apoptosis as a tool to study yeast and human apoptotic regulations. *Frontiers in Physiology* 3. doi:10.3389/fphys.2012.00446

Le Novère, N. (2015). Quantitative and logic modelling of molecular and gene networks. *Nature Reviews Genetics* 16, 146–158. doi:10.1038/nrg3885

Liberzon, A., Birger, C., Thorvaldsdóttir, H., Ghandi, M., Mesirov, J. P., and Tamayo, P. (2015). The molecular signatures database hallmark gene set collection. *Cell systems* 1, 417–425

Martinez-Sanchez, M. E., Mendoza, L., Villarreal, C., and Alvarez-Buylla, E. R. (2015). A minimal regulatory network of extrinsic and intrinsic factors recovers observed patterns of cd4 t cell differentiation and plasticity. *PLOS Computational Biology* 11, e1004324. doi:10.1371/journal.pcbi.1004324

Montagud, A., Traynard, P., Martignetti, L., Bonnet, E., Barillot, E., Zinovyev, A., et al. (2017). Conceptual and computational framework for logical modelling of biological networks deregulated in diseases. *Briefings in Bioinformatics* doi:10.1093/bib/bbx163

Pereira, B., Chin, S.-F., Rueda, O. M., Vollan, H.-K. M., Provenzano, E., Bardwell, H. A., et al. (2016). The somatic mutation profiles of 2,433 breast cancers refines their genomic and transcriptomic landscapes. *Nature Communications* 7. doi:10.1038/ncomms11479

Ríos, O., Frias, S., Rodríguez, A., Kofman, S., Merchant, H., Torres, L., et al. (2015). A boolean network model of human gonadal sex determination. *Theoretical Biology and Medical Modelling* 12. doi:10.1186/s12976-015-0023-0

Rodríguez, A., Sosa, D., Torres, L., Molina, B., Frías, S., and Mendoza, L. (2012). A boolean network model of the FA/BRCA pathway. *Bioinformatics* 28, 858–866. doi:10.1093/bioinformatics/bts036

Saadatpour, A. and Albert, R. (2013). Boolean modeling of biological regulatory networks: A methodology tutorial. *Methods* 62, 3–12. doi:10.1016/j.ymeth.2012.10.012

Schlatter, R., Schmich, K., Vizcarra, I. A., Scheurich, P., Sauter, T., Borner, C., et al. (2009). ON/OFF and beyond - a boolean model of apoptosis. *PLoS Computational Biology* 5, e1000595. doi:10.1371/journal.pcbi.1000595

Stoll, G., Caron, B., Viara, E., Dugourd, A., Zinovyev, A., Naldi, A., et al. (2017). MaBoSS 2.0: an environment for stochastic Boolean modeling. *Bioinformatics* 33, 2226–2228. doi:10.1093/bioinformatics/btx123

Stoll, G., Viara, E., Barillot, E., and Calzone, L. (2012). Continuous time boolean modeling for biological signaling: application of Gillespie algorithm. *BMC Systems Biology* 6, 116. doi:10.1186/1752-0509-6-116

Teschendorff, A. E., Naderi, A., Barbosa-Morais, N. L., and Caldas, C. (2006). PACK: Profile Analysis using Clustering and Kurtosis to find molecular classifiers in cancer. *Bioinformatics (Oxford, England)* 22, 2269–2275. doi:10.1093/bioinformatics/btl174

Tibshirani, R. and Hastie, T. (2007). Outlier sums for differential gene expression analysis. *Biostatistics* 8, 2–8. doi:10.1093/biostatistics/kxl005

Tukey, J. W. (1977). *Exploratory Data Analysis* (Pearson)

Wang, J., Wen, S., Symmans, W. F., Pusztai, L., and Coombes, K. R. (2009). The Bimodality Index: A Criterion for Discovering and Ranking Bimodal Signatures from Cancer Gene Expression Profiling Data. *Cancer Informatics* 7, 199–216

Wang, R.-S., Saadatpour, A., and Albert, R. (2012). Boolean modeling in systems biology: an overview of methodology and applications. *Physical Biology* 9, 055001. doi:10.1088/1478-3975/9/5/055001

Zañudo, J. G. T., Scaltriti, M., and Albert, R. (2017). A network modeling approach to elucidate drug resistance mechanisms and predict combinatorial drug treatments in breast cancer. *Cancer Convergence* 1, 5. doi:10.1186/s41236-017-0007-6
